# Synthesis of a leopolic acid-inspired tetramic acid with antimicrobial activity against multidrug-resistant bacteria

**DOI:** 10.3762/bjoc.14.224

**Published:** 2018-09-24

**Authors:** Luce Mattio, Loana Musso, Leonardo Scaglioni, Andrea Pinto, Piera Anna Martino, Sabrina Dallavalle

**Affiliations:** 1 Department of Food, Environmental and Nutritional Sciences, Università degli Studi di Milano, via Celoria 2, I-20133 Milano, Italy; 2 Department of Veterinary Medicine - Microbiology and Immunology, Università degli Studi di Milano, via Celoria 10, I-20133 Milano, Italy

**Keywords:** antimicrobial activity, multidrug-resistant bacteria, natural products, synthesis, tetramic acid

## Abstract

The increasing emergence of multidrug-resistant pathogens is one of the biggest threats to human health and food security. The discovery of new antibacterials, and in particular the finding of new scaffolds, is an imperative goal to stay ahead of the evolution of antibiotic resistance. Herein we report the synthesis of a 3-decyltetramic acid analogue of the ureido dipeptide natural antibiotic leopolic acid A. The key step in the synthetic strategy is an intramolecular Lacey–Dieckmann cyclization reaction of a linear precursor to obtain the desired 3-alkyl-substituted tetramic acid core. The synthesized analogue is more effective than the parent leopolic acid A against Gram-positive (*Staphylococcus pseudintermedius*) and Gram-negative (*E*. *coli*) bacteria (MIC 8 µg/mL and 64 µg/mL, respectively). Interestingly, the compound shows a significant activity against *Staphylococcus pseudintermedius* strains expressing a multidrug-resistant phenotype (average MIC 32 µg/mL on 30 strains tested). These results suggest that this molecule can be considered a promising starting point for the development of a novel class of antibacterial agents active also against resistant strains.

## Introduction

The treatment of bacterial infections by antibiotics is widely regarded as one of the major achievements of the 20th century. However, the continued emergence of multidrug-resistant bacteria, mainly due to the abuse of antimicrobial molecules (e.g., for treatment of bacterial skin diseases [1]), emphasises the urgent need for novel antibiotic families. In this regard, natural products are privileged compounds, as they possess biologically validated structures, which could become suitable leads in drug discovery [2].

**Figure 1:**
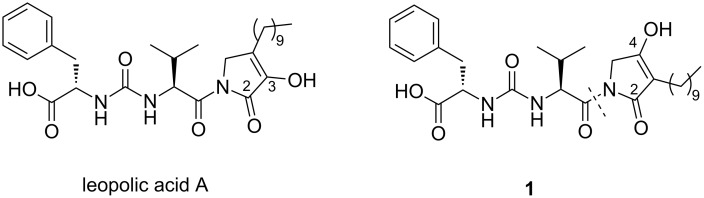
Structures of leopolic acid A and compound **1**.

Recently, our research group reported the first total synthesis of leopolic acid A (Figure 1), a fungal metabolite from a terrestrial-derived *Streptomyces sp*. isolated from the rhizosphere of the plant *Juniperus excelsa* [3–4]. Leopolic acid A is endowed with antibacterial activity against *Staphylococcus aureus* and *Staphylococcus pseudintermedius* with a MIC of 16 μg/mL, and against *Escherichia coli* with a MIC of 128 µg/mL [3–4]. In terms of structural features, this compound contains a 4-decyl-2,3-pyrrolidinedione ring linked to the ureido dipeptide L-Phe-L-Val. The 2,3-pyrrolidinedione ring is a quite unusual skeleton. A limited number of compounds containing this system have been synthesized so far [5–7] and, to the best of our knowledge, natural compounds with a 2,3-pyrrolidinedione nucleus are quite rare [8–11]. The lack of similar compounds may be due to the instability of the 2,3 pyrrolidinedione moiety [12]. Indeed, while developing the total synthesis of leopolic acid A, we encountered several difficulties in the construction of the ring, most of the intermediates being unstable [4].

In light of these results, we intended to investigate the role of the 2,3 pyrrolidinedione ring by replacing it with a more stable isomeric 2,4-pyrrolidinedione moiety. Actually, 2,4 pyrrolidinediones (tetramic acids) have recently attracted considerable attention for their antibacterial, antiviral, antifungal and anticancer activities [13]. More than one hundred of them have been isolated from a variety of natural sources and numerous analogues have been synthesized and studied for their multiple biological activities [13]. For this reason, we planned the synthesis of a leopolic acid A analogue containing the tetramic acid moiety in place of the 2,3-pyrrolidinone ring (compound **1**), while maintaining unchanged all the other structural features of the natural compound. The advantage of this substitution should be a higher stability of the heterocyclic ring, hopefully coupled with an increased activity due to the presence of the tetramic acid core.

In this paper we report the efforts made to develop a synthetic strategy to compound **1**, which may, in principle, have a value in the preparation of various analogues for structure–activity relationship (SAR) studies. The antibacterial activity of compound **1** was tested on *Staphylococcus pseudintermedius* and *Escherichia coli* strains chosen as representative of Gram-positive and Gram-negative bacteria. In particular, we demonstrated the ability of compound **1** to inhibit *Staphylococcus pseudintermedius* strains expressing a multidrug-resistant phenotype.

## Results and Discussion

The instability of most of the *N*-unsubstituted 2,3-pyrrolidinediones prepared for the construction of leopolic acid A [4] forced us to develop a linear synthetic strategy consisting of 11 steps, not amenable for the preparation of analogues. Conversely, compound **1** appears well suited to a convergent synthetic approach based around two fragments, the ureido dipeptide L-Phe-L-Val and the 3-decyltetramic acid core (Figure 1).

**Scheme 1:**
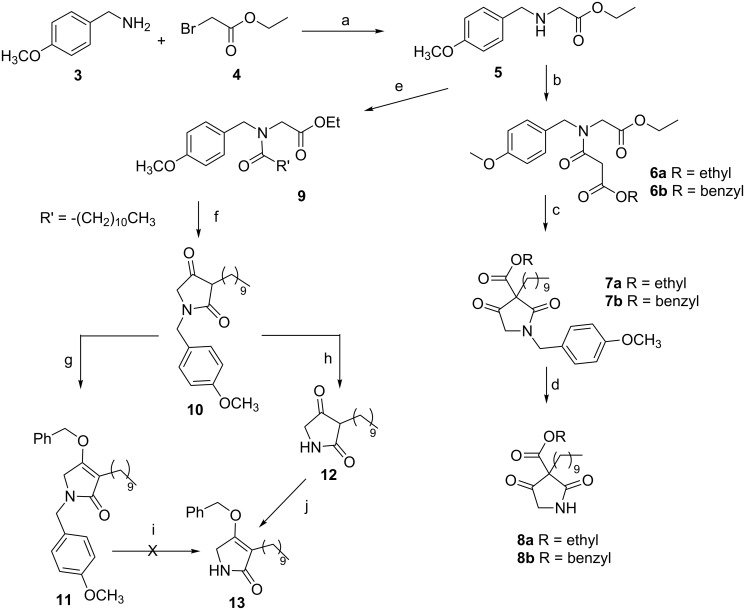
Synthesis of 3-decyltetramic intermediate **13**. Reagent and conditions: a) TEA, THF, 0 °C to rt, 2.5 h, 87%; b) monoethyl malonate (for **6a**), monobenzyl malonate (for **6b**), DCC, DMAP, CH_2_Cl_2_, 0 °C to rt, 24 h (for **6a**), 12 h (for **6b**), **6a**: 80%, **6b**: 83%; c) TBAF, Et_2_O, THF, 1-iododecane, rt, 24 h, **7a**: 22%, **7b**: 30%; d) CAN, CH_3_CN/H_2_O (3:1), 0 °C to rt, 1 h, **8a**: 81%, **8b**: 66%; e) dodecanoyl chloride, TEA, CHCl_3_, 0 °C to rt, 3 h, 90%; f) *t*-BuOK 1 M in THF, THF, reflux, 1.5 h, 65%; g) benzyl tosylate, KHMDS 0.5 M in toluene, crown ether 18-crown-6, THF, 0 °C to rt, 3 h, 35%; h) TFA, 60 °C, 2h; i) CAN, CH_3_CN/H_2_O (3:1), 0 °C to rt, 1h; j) benzyl tosylate, KHMDS 0.5 M in toluene, crown ether 18-crown-6, THF, 0 °C to rt, 2.5 h, 30% over two steps.

Initially, we focused on the synthesis of the 2,4-pyrrolidinedione core. A review of the existing literature on tetramic acids syntheses revealed a considerable amount of papers regarding the preparation of 3-acyltetramic acids [14–18], whereas the synthesis of 3-alkyl-tetramic acids has been considerably less investigated [19–22]. We envisaged that the most straightforward route to the 2,4-pyrrolidinedione system could be a Lacey–Dieckmann cyclization starting from a *N*-acetoacetyl-α-amino ester. Interestingly, the biosynthetic pathways of the tetramic acid scaffold involves Lacey–Dieckmann cyclases [23] or a spontaneous intramolecular Claisen condensation, which occurs in the cytosol. To protect the α-amino ester nitrogen we chose the *p-*methoxybenzyl (PMB) group, easily removable by ceric ammonium nitrate (CAN). *N*-(4-Methoxybenzyl)glycine ethyl ester (**5**) was obtained in 87% yield by reacting 4-methoxybenzylamine (**3**) with bromoacetic acid ethyl ester (**4**) in THF (Scheme 1). The ester **5** was converted into compounds **6a** and **6b** by condensation with monoethyl malonate and monobenzyl malonate, in the presence of DCC and DMAP, in 80% and 83% yield, respectively. Starting from intermediates **6a** and **6b**, treatment with a tetrabutylammonium fluoride solution in diethyl ether at room temperature induced the cyclisation and the formation of an enolate, which was subsequently reacted with 1-iododecane and deprotected with ceric ammonium nitrate to afford derivatives **8a** and **8b**, respectively. Unfortunately, at this stage all attempts to decarboxylate compounds **8a** and **8b** failed [22]. To overcome the problem of decarboxylation, we planned to synthesize the alkyl-substituted tetramic core in one single step by Lacey–Dieckmann cyclisation of ethyl 2-(*N*-(4-methoxybenzyl)dodecanoylamino)acetate (**9**), although this compound does not contain an active methylene group. Thus, compound **5** was acylated with dodecanoyl chloride to obtain compound **9** in 90% yield. As expected, the cyclization reaction was found to be quite troublesome. Several attempts were made using different conditions (TBAF, Et_2_O, rt; NaOEt, EtOH, reflux; NaH, THF, reflux; LDA, THF, −78 °C), but they all were unsuccessful. Finally, we succeeded in preparing intermediate **10** by treatment of compound **9** with potassium *tert*-butoxide (1 M in THF) in THF [24]. The optimisation of reaction conditions, work-up and purification, allowed us to obtain the desired compound in 65% yield.

Before removing the PMB group and installing the ureidodipeptide fragment, we needed to protect the oxygen at C-4 [15]. We selected a benzyl protecting group, as it could be cleaved by catalytic hydrogenation together with the benzyl ester of L-phenylalanine in the ureidodipeptide fragment (see synthesis of compound **20**) by a one-pot reaction. To increase the reaction rate toward *O*-alkylation, we used an aprotic polar solvent like DMF, which weakly solvates the enolates. However, treatment of compound **10** with benzyl bromide and K_2_CO_3_ in DMF gave exclusively the C-3 alkylated derivative. Thus, we considered that a hard leaving group such as a sulfonate should play a key role in favouring *O*-alkylation. Moreover, we selected a base containing potassium as a metal cation, which provides a greater electron density to the nucleophilic enolate, thus favouring *O*-alkylation. Satisfyingly, O-selective alkylation of compound **10** was achieved by deprotonation with KHMDS followed by alkylation with benzyl tosylate in the presence of 18-crown-6 ether [15]. The synthesis of benzyl tosylate was accomplished using benzyl alcohol and freshly recrystallized *p*-toluenesulfonyl chloride in the presence of anhydrous trimethylamine and DMAP, in anhydrous dichloromethane [25].

At this stage, all attempts to obtain the key intermediate **13** removing the *p*-methoxybenzyl group [24,26–28] from **11** failed. Finally, compound **13** was successfully obtained by modifying the sequence of reactions. Deprotection of compound **10** with TFA [24], followed by selective alkylation with benzyl tosylate as previously described, afforded the desired *O*-alkyltetramic acid **13** in 30% yield.

**Scheme 2:**
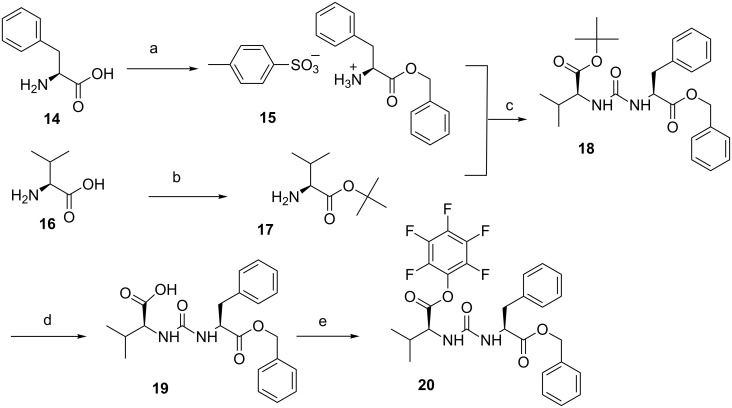
Synthesis of dipeptide L-Phe-L-Val intermediate **20**. Reagents and conditions**:** a) PTSA·H_2_O, benzyl alcohol, toluene, reflux, 10 h, 70%; b) HClO_4_, *tert*-butyl acetate, 0 °C, 1 h, then rt, 20 h, 75%; c) triphosgene, DIEA, DCM, rt, 3 h, 50%; d) trifluoroacetic acid, DCM, rt, 3 h, 95%; e) pentafluorophenol, DCC, EtOAc, 0 °C, 1h, then rt, 3 h, 60%.

The synthesis of the activated ureido fragment was achieved in four steps from suitably protected L-valine and L-phenylalanine. The benzyl protection of L-phenylalanine (**14**) was carried out with PTSA and benzyl alcohol in toluene and the ester **15** was isolated as its *p*-toluensulfonic acid salt by recrystallization with Et_2_O in 70% yield (Scheme 2). L-valine (**16**) was protected as *tert*-butyl ester **17** by using perchloric acid in *t*-BuOAc in 75% yield. The unsymmetrical urea **18** was synthesized using triphosgene at room temperature in 50% yield. The *tert*-butyl ester was easily cleaved by trifluoroacetic acid in DCM at room temperature to furnish the corresponding acid **19** (yield 95%), which was activated by pentafluorophenol, DCC in EtOAc to give the pentafluorophenylester ureido-dipeptide **20** (60%, Scheme 2).

**Scheme 3:**
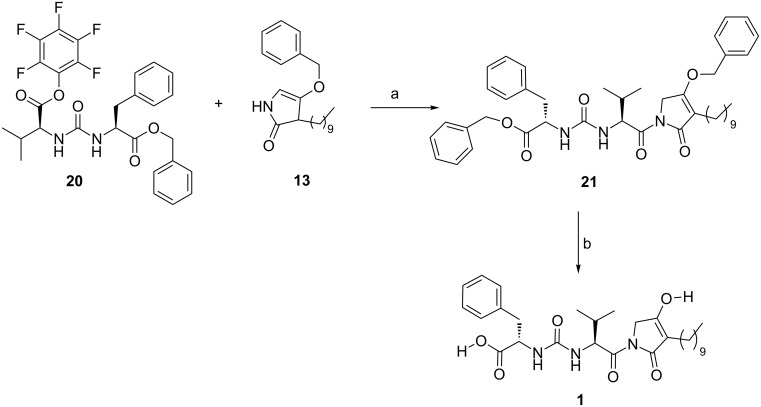
Synthesis of compound **1**. Reagents and conditions**:** a) *n*-BuLi, THF, −60 °C, 220 min, 60%; b) H_2_, Pd/C 10%, AcOEt, rt, 100 min, 72%.

With both key fragments **13** and **20** in hand, we finally accomplished the *N*-acylation reaction using *n*-BuLi in THF at −60 °C [15] in 60% yield. Removal of both protecting groups by catalytic hydrogenation, gave the desired compound **1** in 72% yield (Scheme 3).

Compound **1** was subjected to a preliminary study to evaluate the antimicrobial activity against 80 strains of *Staphylococcus pseudintermedius* and 25 strains of *Escherichia coli*. Bacterial isolates of *S. pseudintermedius* and *E. coli*, previously identified using selective and differential cultural media (e.g., Mannitol Salt Agar; MacConkey Agar, Oxoid, Italy), were isolated on blood agar plates (Tryptic Soy Agar plus 5% defibrinated sheep blood, Microbiol, Italy) to obtain pure cultures [29]. The isolated colonies were used to assess the phenotypic profile of antimicrobial resistance. For this purpose, the Kirby Bauer disk diffusion method was used in accordance to Clinical Laboratory Standards Institute guidelines [30]. All the strains were treated with a panel of antimicrobial molecules belonging to five pharmacological categories: amoxicillin + clavulanic acid, cephalexin, cefovecin, clindamycin, doxycycline, enrofloxacin and marbofloxacin. Only for *S. pseudintermedius* strains, oxacillin was also tested to assess methicillin-resistance (see Table S1, Supporting Information File 1, for details). After incubation, 30 strains of *S. pseudintermedius* revealed resistance phenoptype to three or more pharmacological categories and were considered multidrug resistant (MDR) [31]. MICs (minimum inhibitory concentrations) of compound **1** were evaluated on each bacterial strain (*E. coli* and *S. pseudintermedius* MDR or not) as reported by CLSI guidelines [30,32].

The average MIC values of **1** against 50 *Staphylococcus pseudintermedius* isolates were 8 μg/mL and versus *Escherichia coli* 64 μg/mL, lower than the MICs shown by the parent leopolic acid A (*Staphylococcus pseudintermedius* average MIC 16 μg/mL; *Escherichia coli* average MIC 128 μg/mL) [4]. Interestingly, compound **1** showed a significant activity also against *Staphylococcus pseudintermedius* strains expressing a multidrug-resistant phenotype (average MIC 32 µg/mL on 30 strains tested).

## Conclusion

The development of novel strategies to fight bacterial infections is an imperative goal, mainly due to the increasing number of bacterial strains resistant to a wide spectrum of antibiotics. Aim of this work was the development of a synthetic strategy for obtaining new natural compound-derived scaffolds endowed with increased antimicrobial activity. Attention was focused on 2,4-pyrrolidinedione derivatives, so-called tetramic acids. As part of our search for new tetramic acid containing scaffolds, we have synthesized the 2,4-pyrrolidinone analogue of the natural compound leopolic acid A, by a convergent synthetic strategy. Compound **1** is more effective than the parent leopolic acid A against *Staphylococcus pseudintermedius* and *E*. *coli* strains (MIC 8 µg/mL and 64 µg/mL, respectively) and *Staphylococcus pseudintermedius* strains expressing a multidrug-resistant phenotype (average MIC 32 µg/mL on 30 strains tested). The results confirm that the replacement of the 2,3-pyrrolidinedione core with the tetramic acid nucleus leads to an increase of antimicrobial activity even on MDR strains, thus suggesting that the new scaffold can be considered as a promising candidate for further investigation. Efforts to synthesize analogues of compound **1** to deepen the structure–activity relationship (SAR) study of this novel class of antibacterial agents are underway.

## Supporting Information

File 1 General experimental methods, synthetic procedures and analytical data for the reported compounds, antimicrobial activity evaluation procedures.

File 2 ^1^H NMR and ^13^C NMR spectra of the new compounds; COSY spectra of compounds **1**, **10**; HDMS spectra of compound **1**.
