# Supplementary material for: Synthesis of a leopolic acid-inspired tetramic acid with antimicrobial activity against multidrug-resistant bacteria
Source: Beilstein J Org Chem. 2018 Sep 24;14:2482–7. doi: 10.3762/bjoc.14.224 (PMC6178305; doi:10.3762/bjoc.14.224)
Supplement: File 1 — General experimental methods, synthetic procedures and analytical data for the reported compounds, antimicrobial activity evaluation procedures. [file Beilstein_J_Org_Chem-14-2482-s001.pdf]

Supporting Information File 1

for

Synthesis of a leopolic acid-inspired tetramic acid with antimicrobial activity against multidrug-resistant bacteria

Luce Mattio[1], Loana Musso[1], Leonardo Scaglioni[1], Andrea Pinto[1], Piera Anna Martino[2] and Sabrina Dallavalle*,§,1

Address: [1]Department of Food, Environmental and Nutritional Sciences, Università degli Studi di Milano, via Celoria 2, I-20133 Milano, Italy and [2]Department of Veterinary Medicine - Microbiology and Immunology, Università degli Studi di Milano, via Celoria 10, I-20133 Milano, Italy

Email: Sabrina Dallavalle* - sabrina.dallavalle@unimi.it

*Corresponding author

§Tel. +39 0250316818; Fax +39 0250316801

Table of contents

General experimental methods	S1
Synthetic procedures and analytical data for the reported compounds	S2–S8
Biological activity	S9
Table S1	S9
References	S10

Experimental. General information.

All reagents and solvents were reagent grade or were purified by standard methods before use. Melting points were determined in open capillaries by a SMP3 apparatus and are uncorrected. ^{1}H spectra were recorded on Varian Mercury 300 MHz and Bruker AV600 spectrometers. TMS was used as an internal standard and the chemical shifts were reported in parts per million. The peak patterns are indicated as follows: *s*, singlet; *d*, doublet; *dd*, doublet of doublet; *t*, triplet; *m*, multiplet; *q*, quartet. The coupling constants, *J* are reported in Hertz (Hz). ^{13}C NMR spectra were recorded on Varian 300 MHz and Bruker AV600 spectrometers. Optical rotations were measured with a Perkin Elmer 241 polarimeter. IR spectra were recorded on a Perkin Elmer 1310 spectrophotometer and reported in wave numbers (cm^{-1}). The elemental analyses were recorded with a CARLO ERBA EA 1108 instrument. Mass spectra were recorded on a Q-TOF mass spectrometer (Synapt G2-S HDMS, Waters) equipped with an ESI source. Solvents were routinely distilled prior to use; anhydrous tetrahydrofuran (THF) and ether (Et_2O) were obtained by distillation from sodium-benzophenone ketyl; dry methylene chloride was obtained by distillation from phosphorus pentoxide. All reactions requiring anhydrous conditions were performed under a positive nitrogen flow and all glassware were oven dried and/or flame dried. Isolation and purification of the compounds were performed by flash column chromatography on silica gel 60 (230–400 mesh). Analytical thin-layer chromatography (TLC) was conducted on TLC plates (silica gel 60 F254, aluminium foil). Compounds on TLC plates were detected under UV light at 254 and 365 nm or were revealed spraying with 10% phosphomolybdic acid (PMA) in ethanol.

Compound **5** was prepared as reported in literature. [1]
Compound **6a** was prepared as reported in literature. [2]
Benzyl tosylate was prepared as reported in literature. [3]
Compound **15** was prepared as reported in literature. [4]
Compound **17** was prepared as reported in literature. [5]

***N*-Ethoxycarbonylmethyl-*N*-(4-methoxybenzyl)malonamic acid benzyl ester (6b)**: mono-benzyl malonate (4.1 g, 21.51 mmol, 2 equiv) was added to a solution of compound **5** (2.4 g, 10.75 mmol, 1 equiv) in CH_2Cl_2, followed by a solution of DCC (3.32 g, 16.13 mmol, 1.5 equiv) and DMAP (0.065 g, 0.5 mmol, 0.05 equiv) in CH_2Cl_2 at 0 °C. The solution was stirred 12 h at room temperature. After completion of reaction, the mixture was filtered and the filtrate was washed with water and brine solution. The organic layer was dried over anhydrous Na_2SO_4, filtered and concentrated. The crude product was purified by flash column chromatography (10% EtOAC/hexane) to afford compound **6b** (3.5 g, 83%) as an oil. R_f: 0.5 (EtOAc/hexane 1:4).

^{1}H-NMR (300 MHz, $CDCl_3$) δ: 7.42-7.28 (5H, m); 7.21-7.03 (2H, m); 6.93-6.76 (2H, m); 5.18 (2H, s); 4.55 (2H, s); 4.16 (2H, q, *J* = 7.3 Hz); 4.05 (2H, s); 3.80 (3H, s); 3.63 (2H, s); 1.23 (3H, t, *J* = 7.3 Hz). Anal. C 66.15, H 6.31, N 3.51, calcd for $C_{22}H_{25}NO_6$, Found: C 66.27, H 6.32, N 3.50.

3-Decyl-1-(4-methoxybenzyl)-2,4-dioxopyrrolidine-3-carboxylic acid ethyl ester (7a): to a solution of diester **6a** (200 mg, 0.6 mmol, 1 equiv) in anhydrous diethyl ether (3 mL) tetrabutylammonium fluoride (1M in THF, 1.2 mL, 1.2 mmol, 2 equiv) was added under N_2 atmophere. The reaction was stirred at rt for 2 h, then concentrated in vacuo. The resulting solid was suspended in THF (3 mL), and decyl iodide (0.25 mL, 1.2 mmol, 2 equiv) was added at room temperature. The mixture was stirred for 24 h, then evaporated to dryness. The crude was purified by flash column chromatography (30% EtOAc/Hexane) to afford compound **7a** (102 mg, 22%) as a yellow oil. R_f: 0.5 EtOAc: hexane 3:7

^{1}H-NMR (300 MHz, $CDCl_3$) δ: 7.30-7.18 (2H, m); 6.94-6.79 (2H, m); 4.69 (1H, d, *J* = 13.9 Hz, AB system); 4.53 (1H, d, *J* = 13.9 Hz, AB system); 4.17 (2H, q, *J* = 7.3 Hz); 3.87 (1H, d, *J* = 17.5 Hz, AB system); 3.80 (3H, s, $-OCH_3$); 3.60 (1H, d, *J* = 17.5 Hz); 2.28-2.02 (2H, m); 1.47-1.08 (19H, m); 0.88 (3H, t, *J* = 7.3 Hz). Anal. C 69.58, H 8.64, N 3.25, calcd for $C_{25}H_{37}NO_5$, Found: C 69.48, H 8.65, N 3.25.

3-Decyl-1-(4-methoxybenzyl)-2,4-dioxopyrrolidine-3-carboxylic acid benzyl ester (7b): to a solution of compound **6b** (1.9 g, 4.7 mmol, 1 equiv) in anhydrous diethyl ether (25 mL) tetrabutylammonium fluoride (1M in THF, 9.5 mL, 9.5 mmol, 2 equiv) was added under N_2 atmophere. The reaction was stirred 2 h at rt. The colourless solid was removed by filtration and washed with diethyl ether. The solid was suspended in THF, and decyl iodide (2.5 g, 9.5 mmol, 2 equiv) was added at room temperature. The mixture was stirred for 24 h, then evaporated to dryness. The crude was purified by flash column chromatography (10% EtOAc/Hexane) to afford **7b** (696 mg, 30%). R_f: 0.5 (EtOAc/hexane 1:4).

^{1}H-NMR (300 MHz, $CDCl_3$) δ: 7.46-7.28 (3H, m); 7.28-7.15 (2H, m); 7.14-6.99 (2H, m); 6.81-6.63 (2H, m); 5.15 (2H, s); 4.79 (1H, d, *J* = 14.6 Hz, AB system); 4.44 (1H, d, *J* = 14.6 Hz, AB system); 3.82 (1H, d, *J* = 17.8 Hz, AB); 3.77 (3H, s); 3.57 (1H, d, *J* = 17.8 Hz, AB system); 2.18 (2H, m); 1.48-1.12 (16H, m); 0.88 (3H, t, *J* = 7.2 Hz). Anal. C 72.99, H 7.96, N 2.84, calcd for $C_{30}H_{39}NO_5$, Found: C 72.86, H 7.97, N 2.85.

3-decyl-2,4-dioxopyrrolidine-3-carboxylic acid ethyl ester (8a): to a solution of **7a** (0.23 g, 0.53 mmol) in acetonitrile/water (3:1) cerium ammonium nitrate (0.87 g, 1.59 mmol, 3 equiv) was added at 0 °C. The solution was stirred 1 hour at room temperature. After completion of the reaction, the

mixture was concentrated in vacuo, diluted with EtOAc and washed with water and brine solution. The organic layer was dried over anhydrous Na_2SO_4, filtered and concentrated. The crude product was purified by flash column chromatography (30% EtOAc/Hexane) to afford **8a** (134 mg, 81%) as a yellowish viscous solid. R_f: 0.2 (EtOAC/hexane 3:7)

^{1}H-NMR (300 MHz, $CDCl_3$) δ 4.20 (2H, q, *J* = 7.3 Hz); 4.13 (1H, d, *J* = 17.9 Hz, AB system); 3.84 (1H, d, *J* = 17.9 Hz, AB system); 2.22-2.05 (2H, m); 1.48-1.10 (19H, m). Anal. C 65.57, H 9.39, N 4.50, calcd for $C_{17}H_{29}NO_4$, Found: C 65.46, H 9.38, N 4.51.

3-Decyl-2,4-dioxopyrrolidine-3-carboxylic acid benzyl ester (8b): to a solution of compound **7b** (0.1 g, 0.2 mmol) in acetonitrile : water (3:1), cerium ammonium nitrate (0.3 g, 0.6 mmol, 3 equiv) was added at 0 °C. The solution was stirred 1 h at room temperature. After completion of the reaction, the mixture was diluted with EtOAc and washed with water and brine solution. The organic layer was dried over anhydrous Na_2SO_4, filtered and concentrated. The crude product was purified by flash column chromatography (30% EtOAc/hexane) to afford **8b** (49 mg, 66%) as a colourless liquid. R_f: 0.5 (EtOAc/hexane 1:1).

^{1}H-NMR (300 MHz, $CDCl_3$) δ: 7.43-7.17 (5H, m); 6.95 (1H, brs); 5.16 (2H, s); 4.05 (2H, d, *J* = 18.1 Hz, AB system); 3.81 (2H, d, *J* = 18.1 Hz, AB system); 2.16 (2H, m); 1.46-0.96 (16H, m); 0.88 (3H, t, J = 7.3 Hz).

Anal. C 70.75, H 8.37, N 3.75, calcd for $C_{22}H_{31}NO_4$, Found: C 70.65, H 8.38, N 3.75.

Ethyl 2-(*N*-(4-methoxybenzyl)dodecanamido)acetate (9): to a solution of compound **5** (1.0 g, 4.4 mmol, 1 equiv) and TEA (1.2 mL, 8.8 mmol, 2 equiv) in $CHCl_3$ (10 mL) at 0 °C, dodecanoyl chloride (1.1 g, 8.8 mmol, 1.2 equiv) was added dropwise under nitrogen atmosphere. After 10 minutes at 0 °C, the reaction mixture was stirred 3 hours at room temperature. The reaction was monitored by TLC and after completion, the reaction mixture was diluted with CH_2Cl_2 and was washed with water and brine solution. The organic layer was dried over anhydrous Na_2SO_4, filtered and concentrated under vacuo. The crude product was purified by flash column chromatography (diethylether/hexane 2:3) to afford the desired product **9** (1.63 g, 90%) as a yellow oil. R_f: 0.3 (diethylether/hexane 2:3).

This compound was isolated as a mixture of rotamers in the ratio (2.2:1).

^{1}H-NMR ($CDCl_3$, 300 MHz) mixture of rotamers δ (major): 7.13 (2H, d, *J* = 8.8 Hz); 6.90 (2H, d, *J* = 8.8 Hz); 4.58 (2H, s); 4.18 (2H, q, *J* = 7.3 Hz); 4.02 (2H, s); 3.82 (3H, s); 2.46 (2H, t, *J* = 7.3 Hz); 1.75-1.58 (2H, m); 1.41-1.12 (19H, m); 0.89 (3H, t, J = 7.3 Hz).

^{13}C-NMR ($CDCl_3$, 75 MHz) mixture of rotamers δ (major): 174.1, 169.5, 159.3, 128.1 (×2C), 114. 3(x 2); 61.0; 55.3; 51.6; 46.8; 33.0; 31.9; 29.6 (×2C); 29.5; 29.4 (×3C); 29.3; 25.2; 22.7; 14.1 (×2C).

Anal. C 71.07, H 9.69, N 3.45, calcd for $C_{24}H_{39}NO_4$, Found: C 71.18, H 9.67, N 3.44.

3-Decyl-1-(4-methoxybenzyl)pyrrolidine-2,4-dione (10): To a solution of compound **9** (2 mmol, 810 mg) in anhydrous THF, a solution 1M *t*-BuOK in THF (1.1 equiv, 2.2 mmol, 2.2 mL) was added dropwise at room temperature. The resulting mixture was refluxed under nitrogen for 1.5 h. Then it was cooled to room temperature and poured into cooled 5% aqueous HCl (pH 1–2). THF was evaporated and the aqueous phase was extracted with EtOAc. The combined organic phases were washed with water, dried over anhydrous Na_2SO_4, filtered and concentrated under vacuum.
The residue was triturated with diethyl ether and hexane to give the tetramic acid **15** as a solid (350 mg). The mother liquor was concentrated and purified by flash column chromatography (3:7 EtOAc/hexane) to give the product (110 mg). Yield: 65%. mp: 67-69 °C. R_f: 0.3 (EtOAc : hexane 3:7).

^{1}H-NMR ($CDCl_3$, 600 MHz) δ: 7.19 (2H, d, J = 8.6 Hz); 6.87 (2H, d, J = 8.6 Hz); 4,63 (1H, AB, J = 14.5 Hz); 4.52 (1H, AB system, J = 14.5 Hz); 3.80 (3H, s); 3.70 (1H, AB system, J = 17.8 Hz); 3.57 (1H, dd, J = 17.8 Hz; J = 1.8 Hz); 2.85 (1H, td, J = 5.8 Hz; J = 1.8 Hz); 1.80-1.92 (2H, m); 1.38-1.45 (16H, m); 1.22-1.34 (m); 0.87 (3H, t, J = 7.0 Hz).

^{13}C-NMR ($CDCl_3$, 150 MHz) δ: 207.3; 171.9; 159.4; 129.7 (×2); 127.2; 114.3 (×2); 55.5; 55.3; 50.3; 46.1; 45.4; 31.9; 29.5; 29.5; 29.4; 29.3; 27.3; 26.1; 22.7; 14.1.

Anal. C 73.50, H 9.25, N 3.90, calcd for $C_{22}H_{33}NO_3$, Found: C 73.45, H 9.27, N 3.90.

1-(4-Methoxybenzyl)-4-(benzyloxy)-3-decyl-1*H*-pyrrol-2(5*H*)-one (11): To a stirred solution of compound **10** (200 mg, 0.56 mmol, 1 equiv) in anhydrous THF (2.8 mL), KHMDS 0.5 M in toluene (1.22 mL, 0.62 mmol, 1.1 equiv) was added dropwise under nitrogen, and the mixture was stirred for 10 minutes at 0 °C. Then, benzyl tosylate (213 mg, 0.83 mmol, 1.5 equiv) and crown ether 18-crown-6 (169 mg, 0.64 mmol, 1.15 equiv) were added and the reaction mixture was slowly warmed up to room temperature. After 3 h, the reaction was completed and THF was evaporated in vacuo. The residue was diluted with dichloromethane and was washed with water and brine. The combined organic phases were dried over anhydrous Na_2SO_4, filtered and concentrated under vacuum. The crude was purified by flash column chromatography (30% EtOAc/hexane) to give the product **11** (86 mg, 35%) as a brownish oil. R_f: 0.3 (EtOAc/hexane 3:7).

^{1}H-NMR ($CDCl_3$, 300 MHz) δ: 7.41 – 7.27 (5H, m); 7.14 (2H, d, *J* = 8.8 Hz); 6.85 (2H, d, *J* = 8.8 Hz); 4.98 (2H, s); 4.52 (2H, s); 3.79 (3H, s); 3.71 (2H, s); 2.28 (2H, t, *J* = 7.5 Hz); 1.54-1.44 (m, 2H); 1.42 – 1.16 (m, 14H); 0.88 (t, 3H, J = 6.7 Hz).
Anal. C 77.47, H 8.74, N 3.12, calcd for $C_{29}H_{39}NO_3$, Found: C 77.52, H 8.73, N 3.13.

3-Decylpyrrolidine-2,4-dione (12): A solution of **10** (210 mg, 0.58 mmol, 1 equiv) in trifluoroacetic acid (8.4 mL) was refluxed at 60 °C for 2 h, under nitrogen. Then, trifluoroacetic acid was evaporated in vacuo and the residue was re-dissolved in toluene. The toluene was evaporated and the residue was triturated with a mixture of diethyl ether/hexane (1:9) to give a solid, which was filtered and used directly for the next step without any further purification. R_f: 0.27 (CH_2Cl_2/MeOH 19:1).
^{1}H-NMR ($CDCl_3$, 300 MHz) δ: 10.40 (1H, s); 7.05 (1H, s); 3.64 (2H, s); 2.01 (2H, t, J = 6.7 Hz); 1.40-2.15 (16H, m); 0.86 (3H, t, J = 6.6 Hz).
^{13}C-NMR ($CDCl_3$, 75 MHz) δ: 176.0; 166.9; 105.7; 45.4; 31.8; 29.5 (× 2); 29.4; 29.3; 29.2; 28.4; 22.6; 21.3; 14.4.

4-(Benzyloxy)-3-decyl-1*H*-pyrrol-2(5*H*)-one (13): to a solution of compound **12** (100 mg, 0.41 mmol, 1 equiv) in anhydrous THF (1.9 mL), KHMDS 0.5 M in toluene (0.86 mL, 0.43 mmol, 1.03 equiv) was added dropwise under nitrogen, and the mixture was stirred for 10 minutes at 0 °C. Then, benzyl tosylate (131 mg, 0.5 mmol, 1.2 equiv) and crown ether 18-crown-6 (119 mg, 0.45 mmol, 1.08 equiv) were added and the reaction mixture was slowly warmed up to room temperature and stirred for 2 hours. The solvent was evaporated in vacuo, the residue was diluted with dichloromethane and washed with water and brine. The combined organic phases were dried over anhydrous Na_2SO_4, filtered and concentrated under vacuum. Purification of the crude by flash column chromatography (5% MeOH/CH_2Cl_2) gave the desired product (40 mg) in 30% yield. R_f: 0.45 (CH_2Cl_2/MeOH 19:1).
^{1}H-NMR ($CDCl_3$, 300 MHz) δ: 7.47-7.31 (5H, m); 5.09 (2H, s); 3.94 (2H, s); 2.32-2.19 (2H, m); 1.60-1.43 (2H, m); 1.42-1.15 (14H, m); 0.90 (3H, t, *J* = 6.6 Hz).
^{13}C-NMR ($CDCl_3$, 75 MHz) δ: 176.2; 166.8; 135.9; 129.7; 128.8; 128.5; 128.4; 127.0; 110.8; 71.3; 44.1; 31.9; 29.7; 29.6 (×2); 29.5; 29.3; 28.6; 22.7; 21.9; 14.1.
Anal. C 76.55, H 9.48, N 4.25, calcd for $C_{21}H_{31}NO_2$, Found: C 76.54, H 9.49, N 4.26.

2-[3-(1-Benzyloxycarbonyl-2-phenyl-ethyl)-ureido]-3-methyl-butyric acid *tert*-butyl ester (18): To a stirred solution of triphosgene (180 mg, 0.61 mmol, 0.35 equiv) in dry CH_2Cl_2 (3 mL) a

mixture of **15** (735 mg, 1.73 mmol, 1 equiv) and *N*, *N*-diisopropylethylamine (0.66 mL, 3.81 mmol, 2.2 equiv) in dry CH_2Cl_2 (7.5 mL) was slowly added dropwise at room temperature. The mixture was stirred for five minutes. Then a solution of L-valine *tert*-butyl ester **17** (300 mg, 1.73 mmol, 1 equiv) and *N*,*N*-diisopropylethylamine (0.66 mL, 3.81 mmol, 2.2 equiv) in dry CH_2Cl_2 (7.5 mL) was added in one portion. The reaction was stirred for 3 h under nitrogen, then evaporated to dryness, diluted with ethyl acetate, washed with 10% aqueous $KHSO_4$, 5% aqueous $NaHCO_3$, brine, dried over Na_2SO_4. After filtration and concentration to dryness, the crude was purified by flash column chromatography (25% EtOAc/hexane) to give 350 mg (50%) of the product **18** as a grey solid. m.p.: 110-112 °C. $[\alpha]_D^{25}$: +4.6. (*c* , 0.5, CH_2Cl_2). R_f: 0.4 (EtOAc : hexane 1:3)

^{1}H-NMR ($CDCl_3$, 300 MHz) mixture of rotamers δ (major): 7.38-7.24 (5H, m); 7.23-717 (3H, m); 7.06-6.97 (2H, m); 5.15 (1H, d, J = 12.2 Hz); 5.08 (1H, brs); 5.06 (1H, d, *J* = 12.2 Hz); 4.96 (1H, brs); 4.87-4.77 (1H, m); 4.28 (1H, dd, *J* = 4.3); 3.14-3.01 (2H, m); 2.12-2.03 (1H, m); 1.44 (9H, s); 0.90 (3H, d, J = 6.9); 0.82 (3H, d, J = 6.9).

^{13}C-NMR ($CDCl_3$, 75 MHz) mixture of rotamers δ (major): 172.5; 172.3; 156.8; 136.0; 135.2; 129.4 (× 2); 128.5 (× 2); 128.46 (× 2); 128.4 (× 2); 128.3; 126.8; 81.7, 67.0; 58.3; 54.1; 38.6; 31.6; 28.0 (× 3); 18.9; 17.5.

Anal. C 68.70, H 7.54, N 6.16, calcd for $C_{26}H_{34}N_2O_5$, Found: C 68.65, H 7.52, N 6.17.

2-[3-(1-Benzyloxycarbonyl-2-phenyl-ethyl)-ureido]**-3-methyl-butyric acid (19)**: to a solution of compound **18** (120 mg, 0.26 mmol) in dry CH_2Cl_2 (7.9 mL), trifluoroacetic acid (2.6 mL) was added at 0 °C under nitrogen and the mixture was stirred at room temperature for 3 h. Trifluoroacetic acid was removed in vacuo and the residue was re-dissolved in toluene (5 mL). The toluene was evaporated and the operation was repeated two times. The crude was treated with hexane to give the product **19** as a grey solid (100 mg, 95%). m.p.:178-180 °C. $[\alpha]_D^{25}$: -2.4. (*c* , 0.5, MeOH). R_f: 0.2 (CH_2Cl_2: MeOH: AcOH 19: 1: 0.1)

^{1}H-NMR (CD_3OD, 300 MHz) mixture of rotamers δ (major): 7.38-7.25 (5H, m); 7.24-7.17 (3H, m); 7.14-7.07 (2H, m); 5.12 (1H, d, AB system, -O-CH-H-Ph, J = 12.3 Hz); 5.08 (1H, d, AB system, -O-CH-H-Ph, J = 12.3 Hz); 4.64–4.56 (1H, m); 4.20–4-14 (1H, m); 3.12–2.94 (2H, m); 2.19–2.03 (1H, m); 0.98-0.85 (6H, m).

^{13}C-NMR (CD_3OD, 75 MHz) mixture of rotamers δ (major): 174.4; 172.3; 158.6; 136.4; 135.6; 129.0 (×2); 128.1 (×2); 128.0 (×4); 127.9; 126.4; 66.5; 57.9; 54.4; 37.7; 30.6; 18.2; 16.5.

Anal. C 66.32, H 6.58, N 7.03, calcd for $C_{22}H_{26}N_2O_5$, Found: C 66.40, H 6.59, N 7.04.

2-[3-(1-Benzyloxycarbonyl-2-phenyl-ethyl)-ureido]-3-methyl-butyric acid pentafluorophenyl ester (20): a solution of **19** (90 mg, 0.22 mmol, 1 equiv), pentafluorophenol (46.5 mg, 0.25 mmol,

1.13 equiv), DCC (52 mg, 0.25 mmol, 1.13 equiv) in EtOAc (1.6 mL) was stirred at 0 °C for 1h, then 3h at room temperature. The resultant mixture was filtered and the filtrate was concentrated under reduced pressure. The crude was treated with hexane and Et_2O to give the product **20** as a white solid (74 mg, 60%). m.p.: 93-95 °C. $[\alpha]_D^{25}$ = +6.8 (*c*, 0.5, CH_2Cl_2). R_f: 0.3 (ETP/EtOAc 85:15)

^{1}H-NMR ($CDCl_3$, 300 MHz) δ: 7.41–7.27 (5H, m); 7.23–7.15 (3H, m); 7.03–6.95 (2H, m); 5.17 (1H, AB system, *J* = 12.2 Hz); 5.10 (1H, d, *J* = 8.1 Hz); 5.09 (1H, AB system, *J* = 12.2 Hz), 5.04 (1H, d, *J* = 9.0 Hz); 4.85 (1H, dt, *J* = 8.1 Hz; *J* = 5.6 Hz); 4.77 (1H, dd, *J* = 9.0 Hz, *J* = 4.8 Hz); 3.09 (2H, d, *J* = 5.6 Hz); 2.38–2.23 (m, 1H); 1.04 (3H, d, *J* = 6.9 Hz); 0.97 (3H, d, *J*= 6.9 Hz).

^{13}C-NMR ($CDCl_3$, 75 MHz) δ: 172.6; 169.4; 156.4; 142.7; 141.3; 139.5; 138.0; 136.3; 135.8 (×2); 135.0; 129.4 (×2); 128.6 (×2); 128.5 (×3); 128.4 (×2); 126.9; 67.2; 58.1; 54.2; 38.4; 31.2; 18.8; 17.4.

2-{3-[1-(4-Benzyloxy-2-oxo-3-decyl-2,5-dihydropyrrole-1-carbonyl)-2-methylpropyl]-ureido}-3-phenylpropionic acid benzyl ester (21): to a stirred solution of **13** (53 mg, 0.161 mmol, 1 equiv) in THF (3 mL) at −60 °C, under nitrogen, a solution of *n*-BuLi 1.6 M in hexane (60 µl) was added dropwise. The mixture was stirred for 20 minutes. Then, a solution of **20** (100 mg, 0.177 mmol, 1.1 equiv) in THF (2 mL) was added dropwise over 20 minutes. The mixture was allowed to stir for further 3 hours. The temperature was allowed to rise to −45 °C and the mixture was quenched with AcOH (60 µl) and then the mixture was concentrated under reduced pressure. The crude was purified by flash column chromatography (acetone/hexane 3:17) to give the product (67 mg, 60%) as a white solid. m.p.: 131-133°C. $[\alpha]_D^{25}$+9 (*c* , 0.5, CH_2Cl_2). R_f: 0.3 (hexane/ acetone 4:1).

^{1}H-NMR ($CDCl_3$, 600 MHz) δ: 7.45–7.30 (8H, m); 7.23-7.14 (4H, m); 7.11-7.06 (1H, m); 7.00-6.90 (2H, m); 6.64 (1H, brs); 6.14 (1H, d, *J* = 8.4 Hz); 5.90 (1H, d, *J* = 8.8 Hz); 5.16-5.21 (1H, m); 5.06 (2H, AB system, *J* = 11.9 Hz); 4.98 (2H, AB system, *J* = 12.5 Hz); 4.07 (1H, AB system, *J* = 17.9 Hz); 3.38 (1H, AB system, *J* = 17.9 Hz); 3.15 (1H, dd, *J* = 13.6 Hz; *J* = 3.7 Hz); 2.94 (1H, dd, *J* = 13.6 Hz; *J* = 5.3 Hz); 2.17 – 2.12 (3H, m); 1.55 – 1.45 (14H, m); 1.35-1.20 (2H, m); 1.05 (3H, d, *J* = 6.8 Hz); 0.88 (3H, t, *J* = 7.2 Hz); 0.78 (3H, d, *J* = 6.8 Hz).

^{13}C-NMR ($CDCl_3$, 125 MHz) δ: 173.8; 172.5; 170.3; 169.8; 157.5; 135.8; 135.4; 135.0; 129.8; 129.1 (× 2); 129.0 (× 2); 128.8 (× 2); 128.6 (× 2); 128.5; 128.2 (× 2); 127.5 (× 2); 127.2; 110.6; 72.0; 67.0; 57.6; 53.6; 45.5; 39.6; 32.1; 31.2; 29.8; 29.7; 29.5; 29.4; 28.1; 22.8; 21.6; 19.9; 16.5; 14.2.

2-{3-[1-(3-Decyl-4-hydroxy-2-oxo-2,5-dihydropyrrole-1-carbonyl)-2-methylpropyl]-ureido}-3-phenylpropionic acid (1): in a similar manner as described in [6], to a stirred solution of **21** (40 mg, 0.056 mmol) in EtOAc (5 mL) a catalytic amount of Pd/C 10% (8 mg, 20% w/w) was added. The reaction mixture was evacuated and flushed with hydrogen gas (3 times) and stirred at room temperature under hydrogen atmosphere for 100 minutes at rt. After completion, the reaction mixture was filtered through a short pad of celite and the pad was washed with EtOAc (3 × 5 mL). The filtrate was concentrated in vacuo to give the pure product **1** as a white solid (21 mg, 72%). m.p.: 134°C dec. $[\alpha]_D^{25}$+36 (*c*, 0.5, MeOH). R_f =0.44 (MeOH/DCM 1:9).

^{1}H-NMR (CD_3OD_3, 600 MHz) δ: 7.30-7.16 (5H, m); 5.51 (1H, brs); 4.54 (1H, t, J = 5.9 Hz); 4.17 (1H, AB system, J = 17.7 Hz); 4.05 (1H, AB system, J = 17.7 Hz); 3.09 (1H, dd, J = 13.7 Hz; J = 5.3 Hz); 2.99 (1H, dd, J = 13.7 Hz; J = 6.9 Hz); 2.16 (3H, m); 1.43-1.49 (2H, m); 1.25 – 1.36 (14H, m); 1.02 (3H, d, J = 6.9 Hz); 0.89 (3H, t, J = 6.9 Hz); 0.85 (3H, d, 6.9 Hz).

^{13}C-NMR (CD_3OD_3, 125 MHz) δ: 175.6; 174.0; 173.3; 171.9, 160.2, 138.2; 130.6 (× 2); 129.3 (× 2); 127.7; 107.6; 58.9; 55.5; 49.4 (overlapped to solvent signal CD_3OD); 39.2; 33.1; 31.7; 30.7 (× 2); 30.5 (× 2) ; 30.4; 29.1; 23.7; 21.9; 20.2; 16.7; 14.4.

HDMS**:** (ES^-) calculated for $C_{29}H_{42}N_3O_6$ $(M-H)^-$ 528.3074, Found: 528.3084.

Biological activity. MIC determination.

In a similar manner as described in [6], MIC were determined for each bacterial strains (*Staphylococcus pseudintermedius* and *Escherichia coli*) using the broth microdilution method, according to the Clinical and Laboratory Standards Institute (CLSI) reference methods [7]. The drug solutions were prepared in DMSO to obtain a initial concentration of 1 mg/mL. Overnight cultures were prepared and adjusted to a density of 0.5 Mc Farland scale; 80 strains of *Staphylococcus pseudintermedius* and 25 strains of *Escherichia coli* were used. For the tests 96-wells sterile microtiter plates were used and compounds dilutions (1:2) were performed in Mueller Hinton Broth (Thermo Fisher Scientific, Italy). Negative and positive control were included. After incubation at 37 °C for 24 h, the MIC was determined as the lowest concentration of compound at which there was visible growth.

Table S1: contents in μg for each antimicrobial disk used for Kirby–Bauer test

**Antimicrobials**	**μg/disk**
Amoxicillin + clavulanic acid	30
Cephalexin	30
Cefovecin	30
Clindamycin	2
Doxycycline	30
Enrofloxacin	5
Marbofloxacin	5
Oxacillin	5

References

[1] Williams, R.M.; Glinka, T.; Kwast, E.; Coffman, H.; Stille, J. K. *J. Am. Chem. Soc*. **1990**, *112*, 808-21, DOI: 10.1021/ja00158a048.

[2] Wood, J. L.; Stoltz, B. M.; Dietrich, H.-J.; Pflum, D. A.; Petsch, D. T. *J. Am. Chem. Soc*. **1997**, *119*, 9641-9651, DOI: 10.1021/ja9713035.

[3] Katakawa, K.; Yonenaga, D.; Terada, T.; Aida, N.; Sakamoto, A.; Hoshino, K.; Kumamoto, T. *Heterocycles*, **2014**, *88*, 817-825, doi: 10.3987/COM-13-S(S)66.

[4] Thaqi, A.; McCluskey, A.; Scott J. L. *Tetrahedron Letters*, **2008**, *49*, 6962-6964, doi.org/10.1016/j.tetlet.2008.09.027.

[5] Chen, H.; Feng, Y.; Xu, Z.; Ye, T. The total synthesis and reassignement of stereochemistry of dragonamide *Tetrahedron,* **2005**, *61*, 11132-11140, doi.org/10.1016/j.tet.2005.09.040.

[6] Dhavan, A. A.; Kaduskar, R. D.; Musso, L.; Scaglioni, L; Martino, P. A.; Dallavalle S. *Beilstein J. Org. Chem.* **2016**, *12*, 1624–1628. doi:10.3762/bjoc.12.159

[7] Clinical and Laboratory Standards Institute 2015. Performance Standard for Antimicrobial Disk and Dilution Susceptibility Tests for Bacteria Isolated from Animals, 3rd Edition CLSI VET01S (Wayne, Pennsylvannia, Clinical Laboratory and Standards Institute), 128, **2015**.
