# Supplementary material for: Synthesis of a leopolic acid-inspired tetramic acid with antimicrobial activity against multidrug-resistant bacteria
Source: Beilstein J Org Chem. 2018 Sep 24;14:2482–7. doi: 10.3762/bjoc.14.224 (PMC6178305; doi:10.3762/bjoc.14.224)
Supplement: File 2 — 1H NMR and 13C NMR spectra of the new compounds; COSY spectra of compounds 1, 10; HDMS spectra of compound 1. [file Beilstein_J_Org_Chem-14-2482-s002.pdf]

Supporting Information- File 2

for

Synthesis of a leopolic acid-inspired tetramic acid with antimicrobial activity against multidrug-resistant bacteria

Luce Mattio[1], Loana Musso[1], Leonardo Scaglioni[1], Andrea Pinto[1], Piera Anna Martino[2] and Sabrina Dallavalle*[,§,1]

Address: [1]Department of Food, Environmental and Nutritional Sciences, Università degli Studi di Milano, via Celoria 2, I-20133 Milano, Italy and [2]Department of Veterinary Medicine - Microbiology and Immunology, Università degli Studi di Milano, via Celoria 10, I-20133 Milano, Italy

Email: Sabrina Dallavalle* - sabrina.dallavalle@unimi.it

*Corresponding author

[§]Tel. +39 0250316818; Fax +39 0250316801

^{1}H NMR and ^{13}C NMR spectra of the new compounds; COSY spectra of compounds 1, 10; HDMS spectra of compound 1

Table of contents

^{1}H-NMR Spectrum of compound **9** S1

^{13}C-NMR Spectrum of compound **9** S2

^{1}H-NMR Spectrum of compound **10** S3

^{13}C-NMR Spectrum of compound **10** S4

COSY Spectrum of compound **10** S5

^{1}H-NMR Spectrum of compound **11** S6

^{1}H-NMR Spectrum of compound **12** S7

^{13}C-NMR Spectrum of compound **12** S8

^{1}H-NMR Spectrum of compound **13**	S9
^{13}C-NMR Spectrum of compound **13**	S10
^{1}H-NMR Spectrum of compound **18**	S11
^{13}C-NMR Spectrum of compound **18**	S12
^{1}H-NMR Spectrum of compound **19**	S13
^{13}C-NMR Spectrum of compound **19**	S14
^{1}H-NMR Spectrum of compound **20**	S15
^{13}C-NMR Spectrum of compound **20**	S16
^{1}H-NMR Spectrum of compound **21**	S17
^{13}C-NMR Spectrum of compound **21**	S18
^{1}H-NMR Spectrum of compound **1**	S19
^{13}C-NMR Spectrum of compound **1**	S20
COSY Spectrum of compound **1**	S21
MS Spectrum of compound **1**	S22

^{1}H-NMR (300 MHz, $CDCl_3$) spectrum of compound **9** (mixture of rotamers)

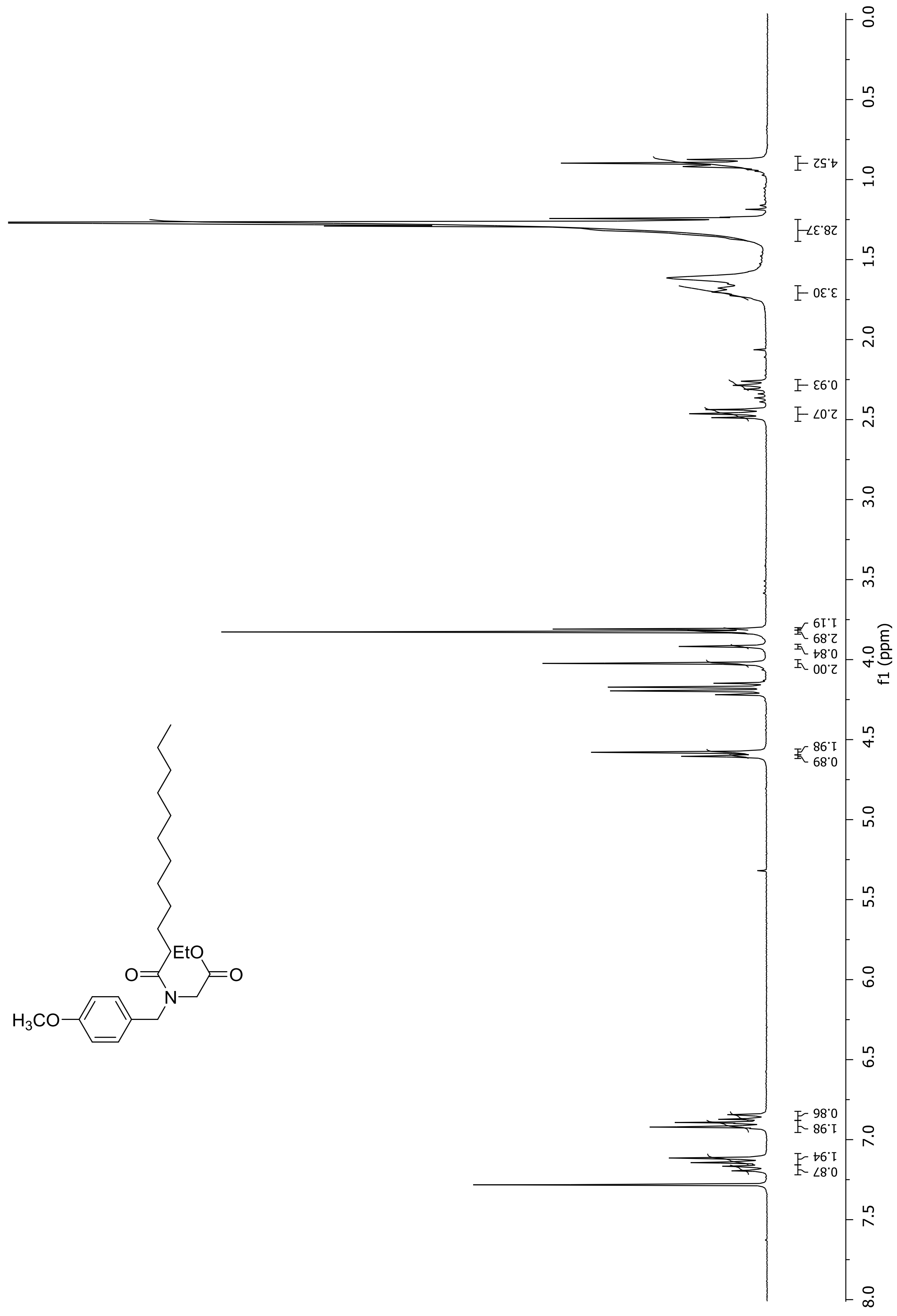

^{13}C-NMR (75 MHz, CDCl$_3$) spectrum of compound **9** (APT) (mixture of rotamers)

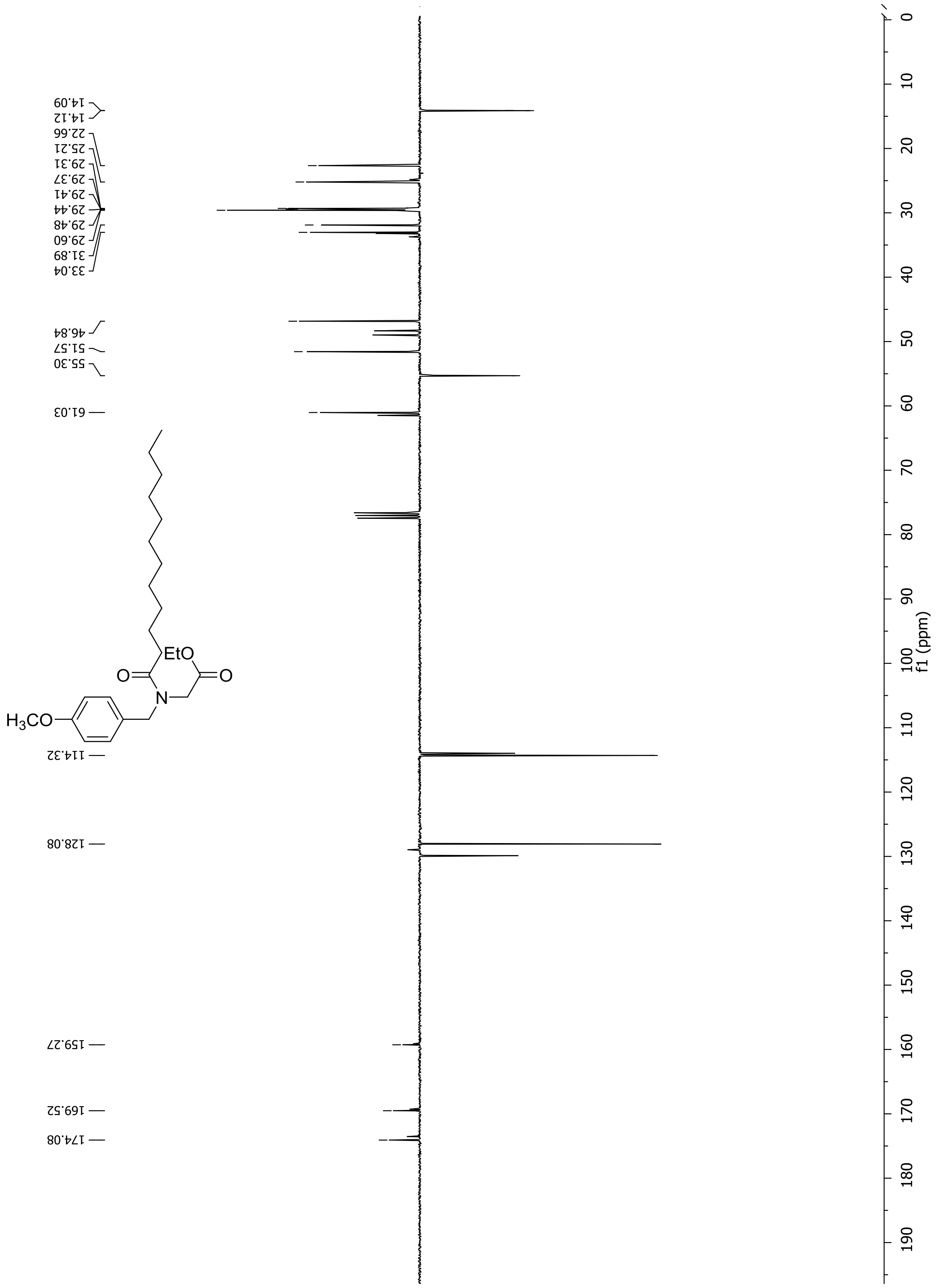

^{1}H-NMR (600 MHz, $CDCl_3$) spectrum of compound **10**

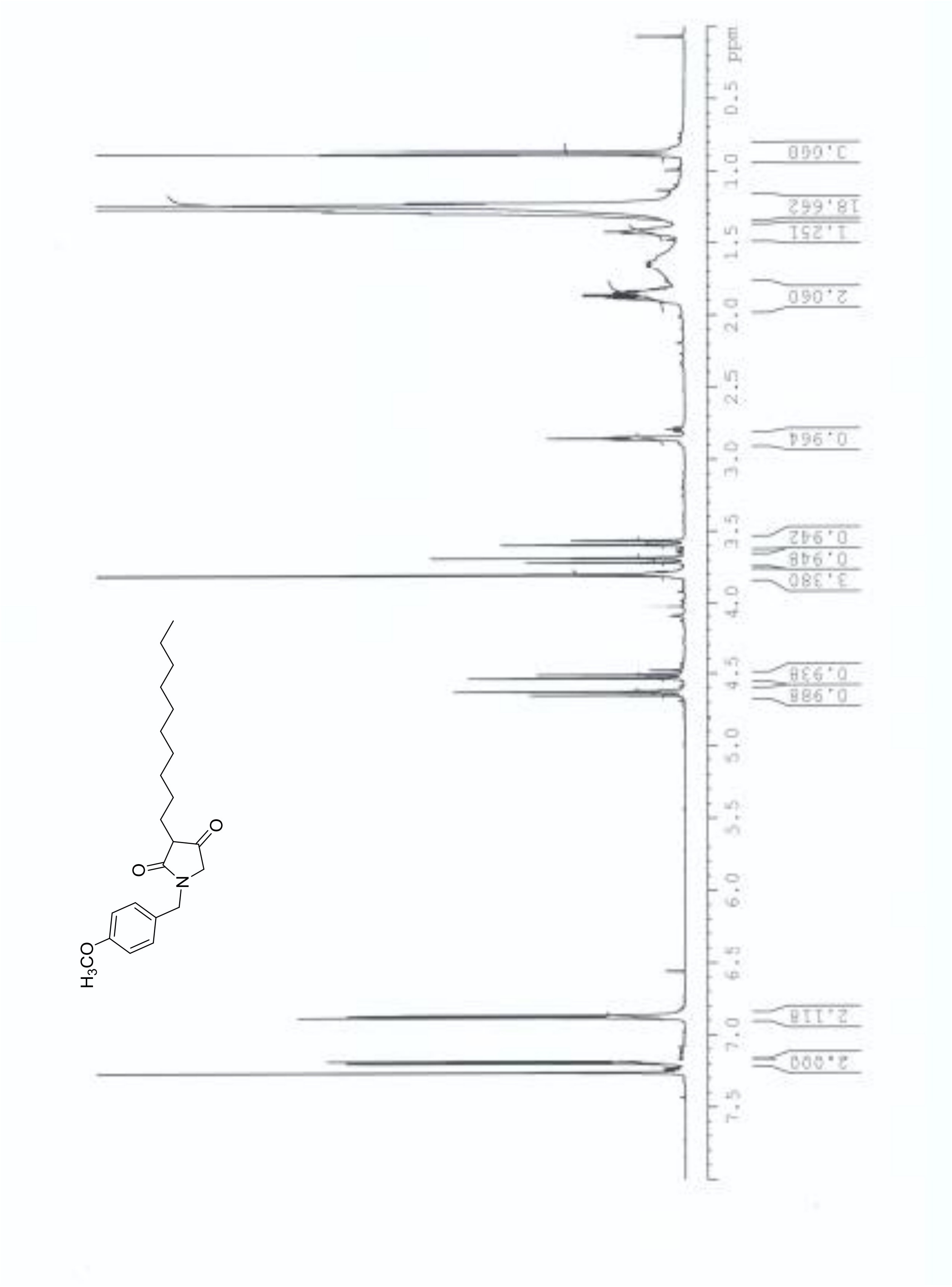

^{13}C-NMR (150 MHz, $CDCl_3$) spectrum of compound **10**.

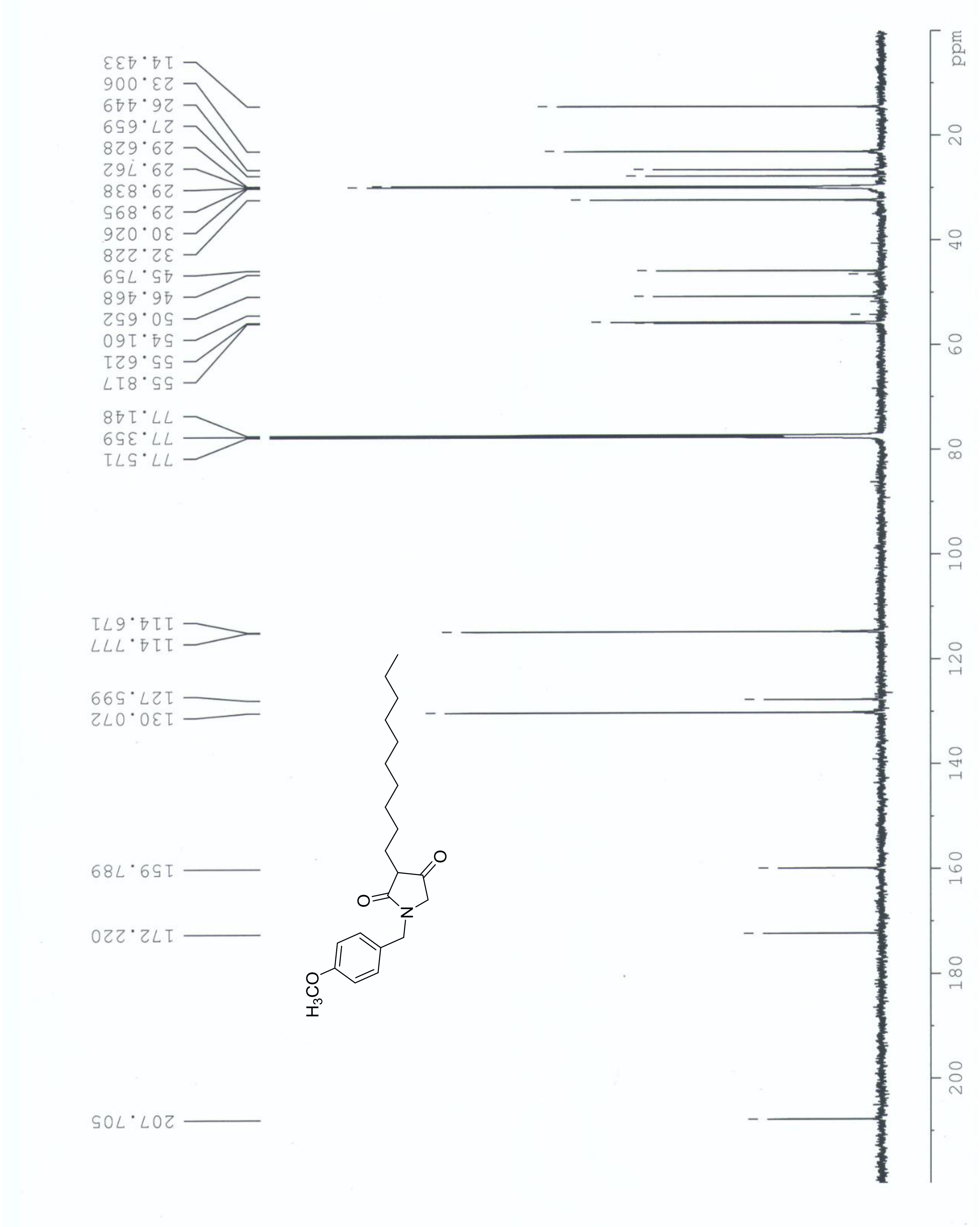

COSY (600 MHz, $CDCl_3$) spectrum of compound **10**.

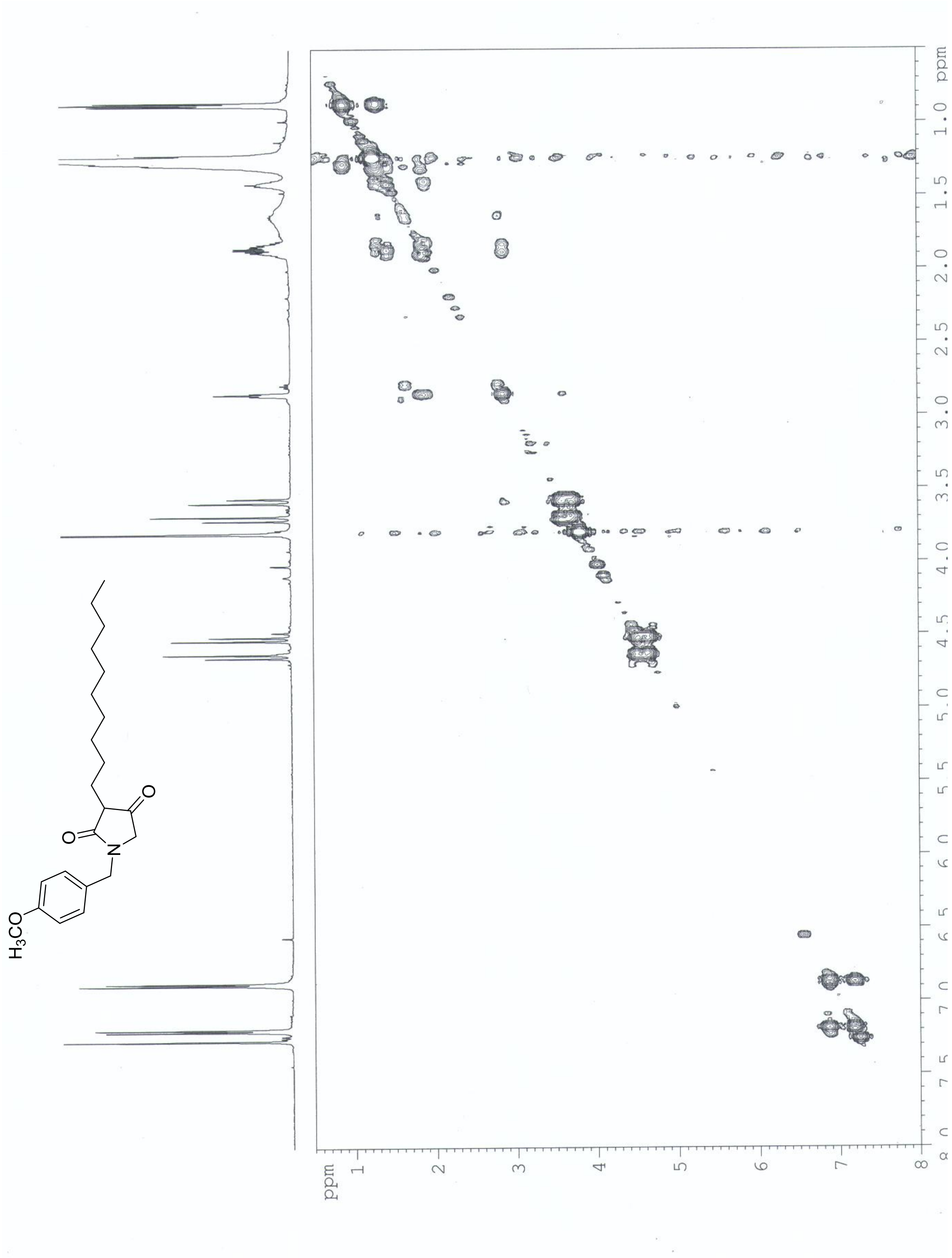

^{1}H-NMR (300 MHz, $CDCl_3$) spectrum of compound **11**.

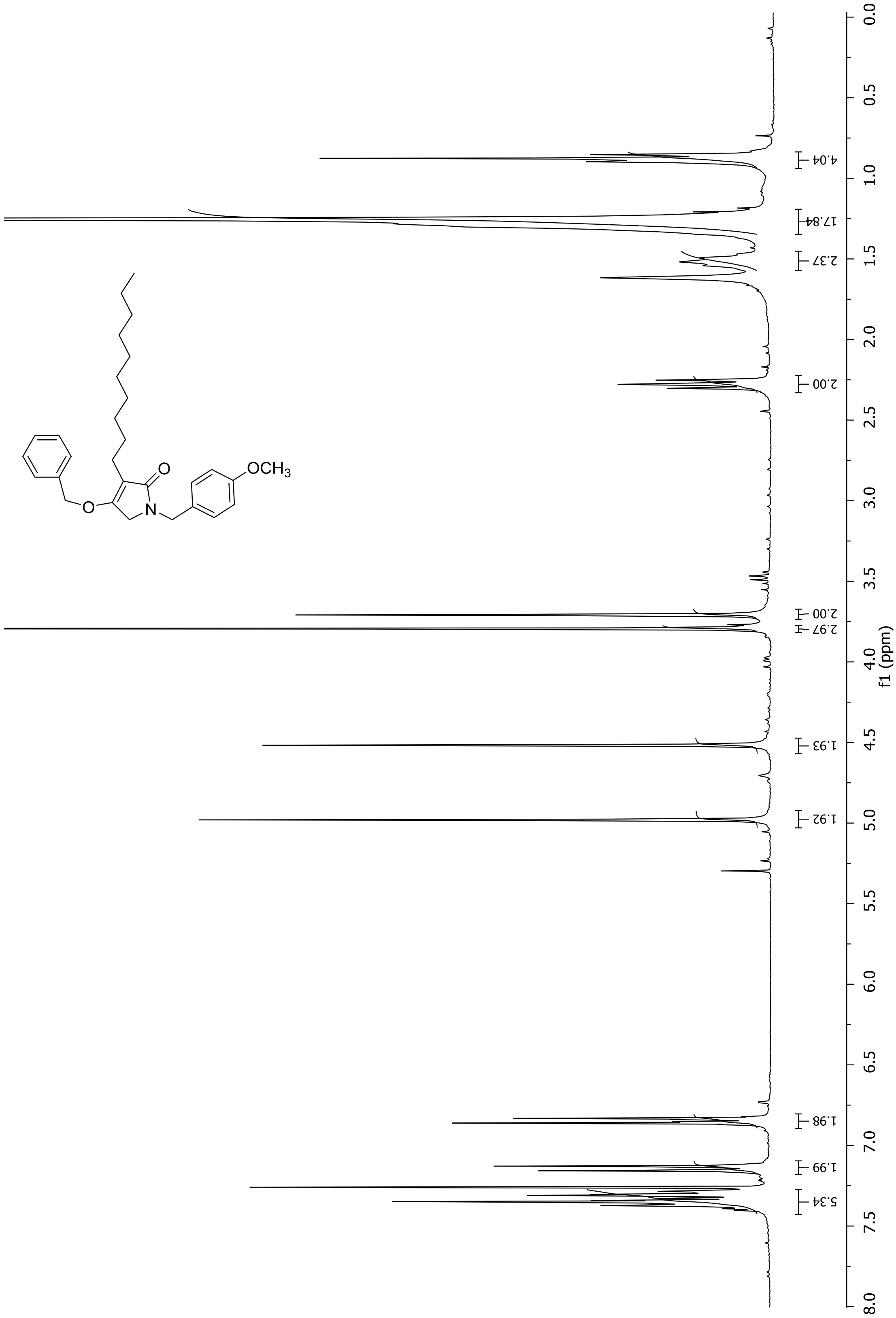

^{1}H-NMR (300 MHz, d_6-DMSO) spectrum of compound **12**.

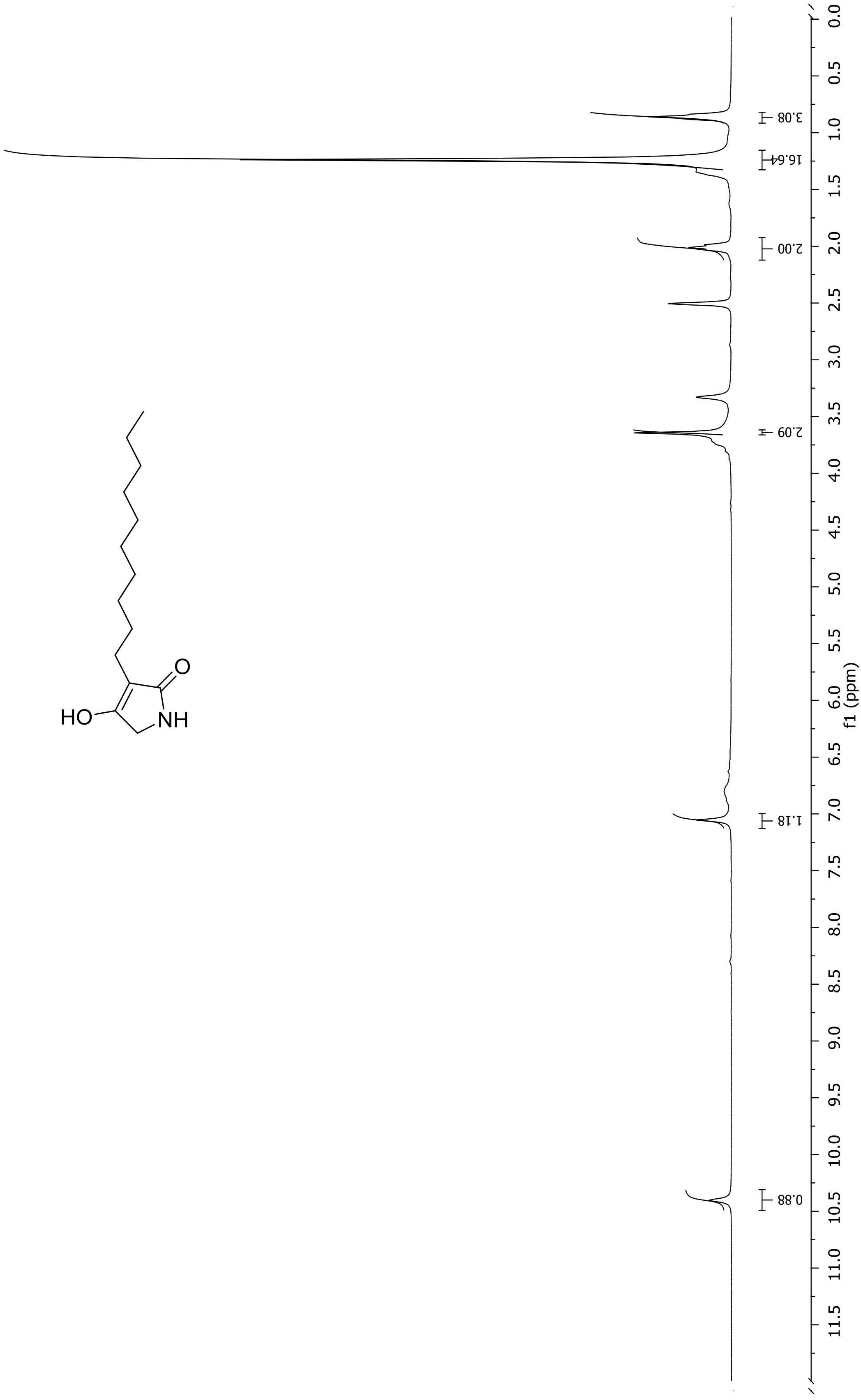

^{13}C-NMR (75 MHz, d_6-DMSO) spectrum of compound **12** (APT)

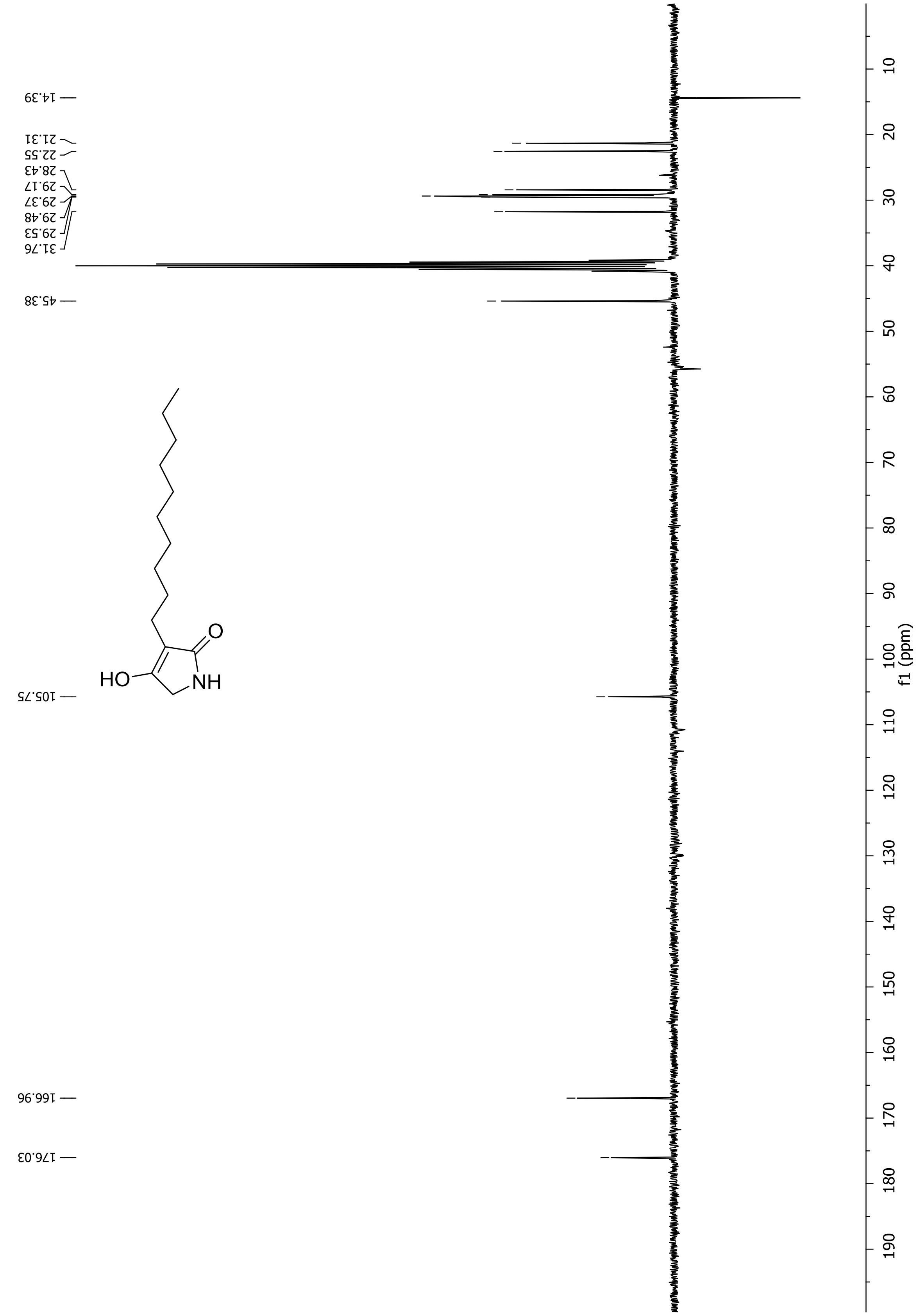

^{1}H-NMR (300 MHz, $CDCl_3$) of compound **13**.

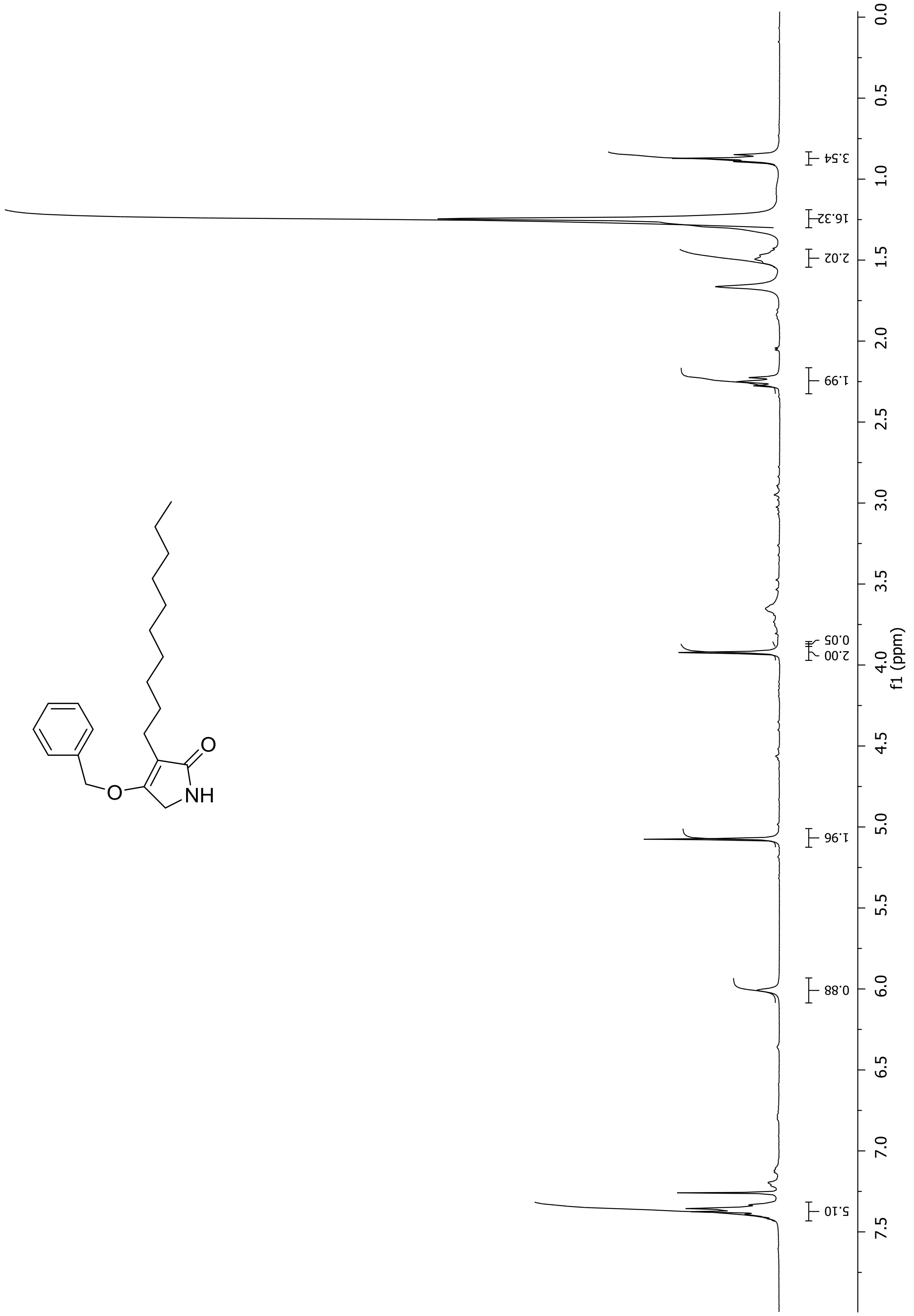

^{13}C-NMR (75 MHz, $CDCl_3$, APT) spectrum of compound **13**.

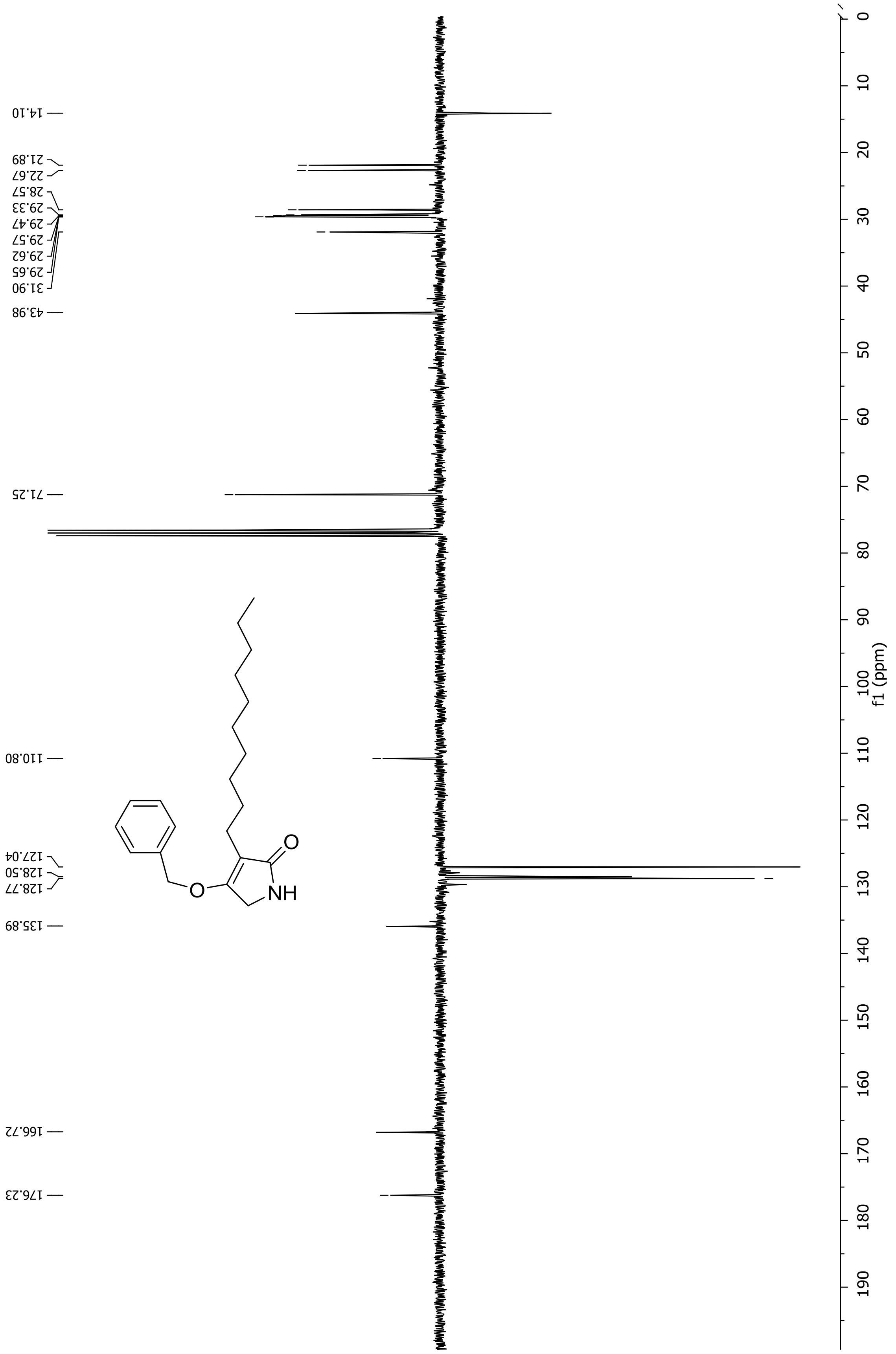

^{1}H-NMR (300 MHz, $CDCl_3$) spectrum of compound **18** (mixture of rotamers).

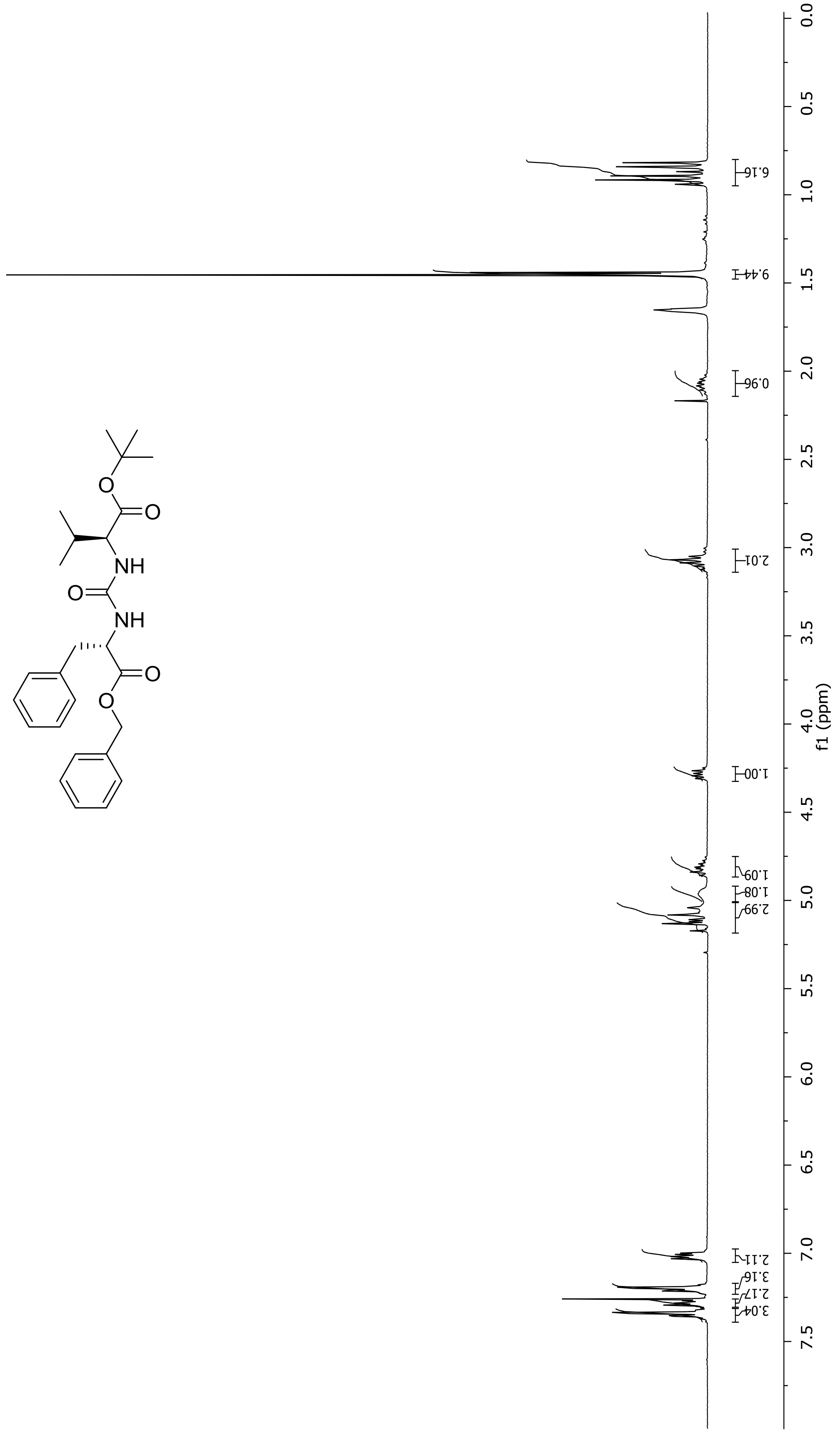

^{13}C-NMR (75 MHz, $CDCl_3$) APT spectrum of compound **18** (mixture of rotamers)

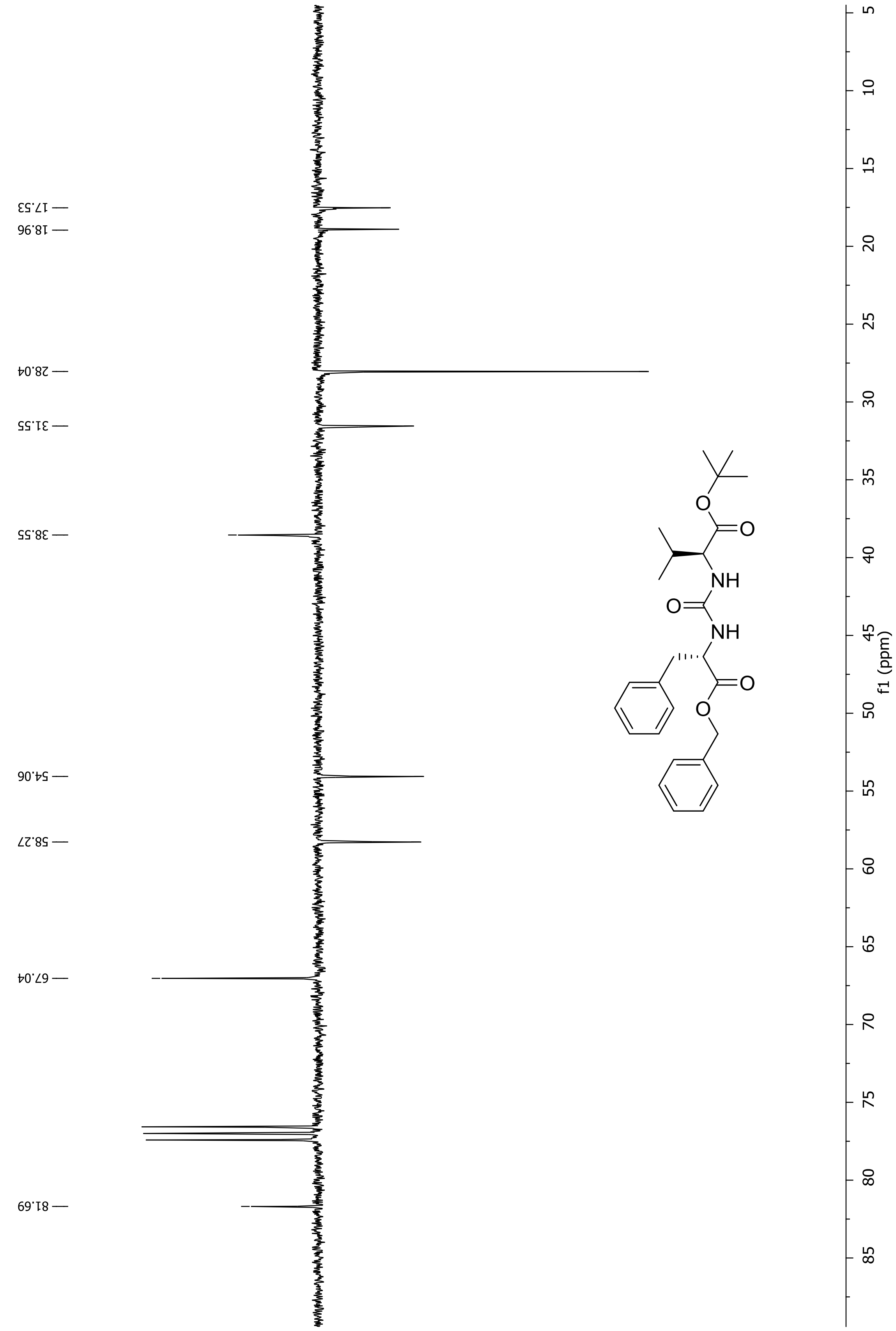

^{1}H-NMR (300 MHz, CD_3OD) spectrum of compound **19** (mixture of rotamers)

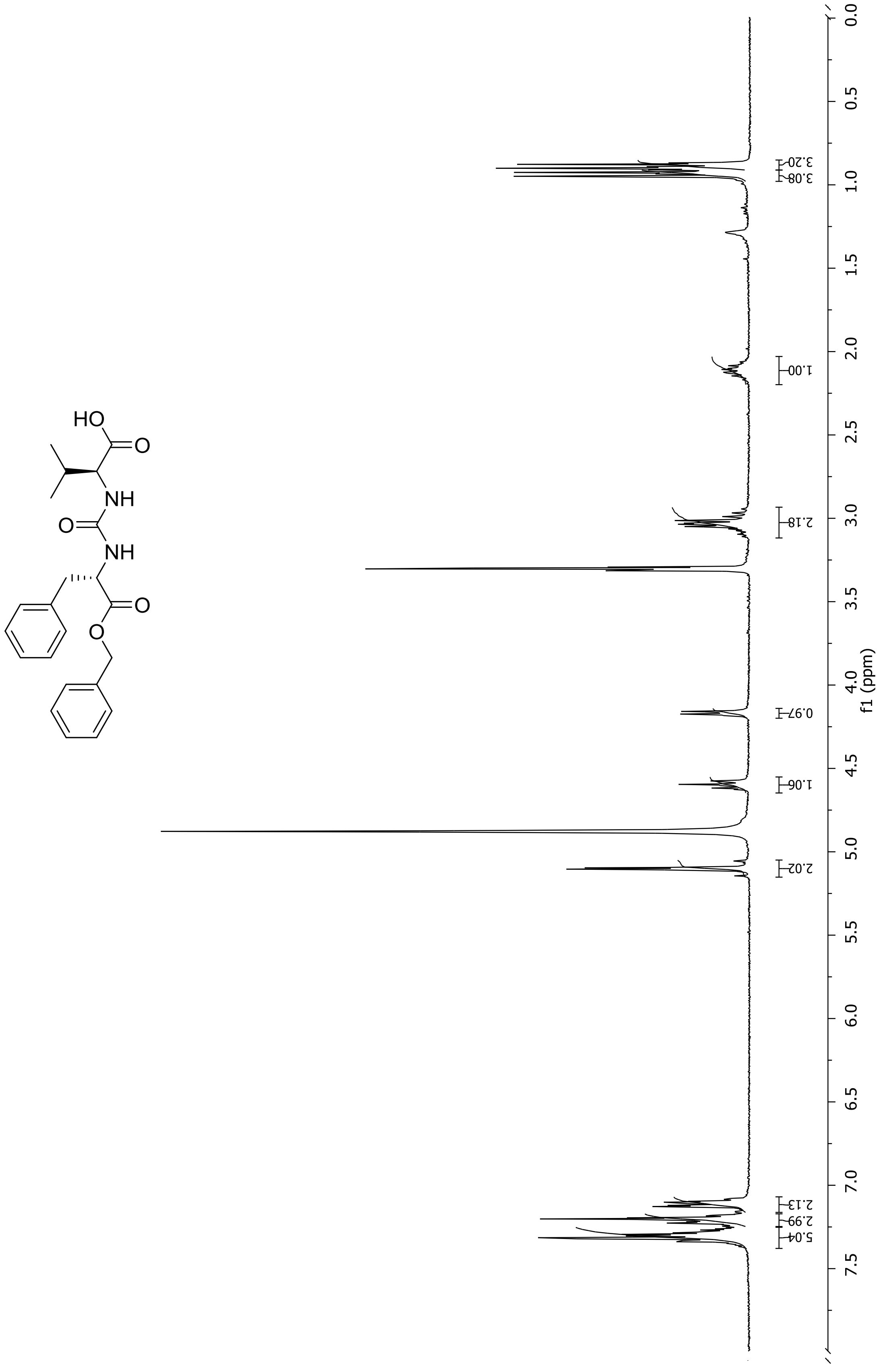

^{13}C-NMR (75 MHz, CD_3OD) spectrum of compound **19** (APT) (mixture of rotamers)

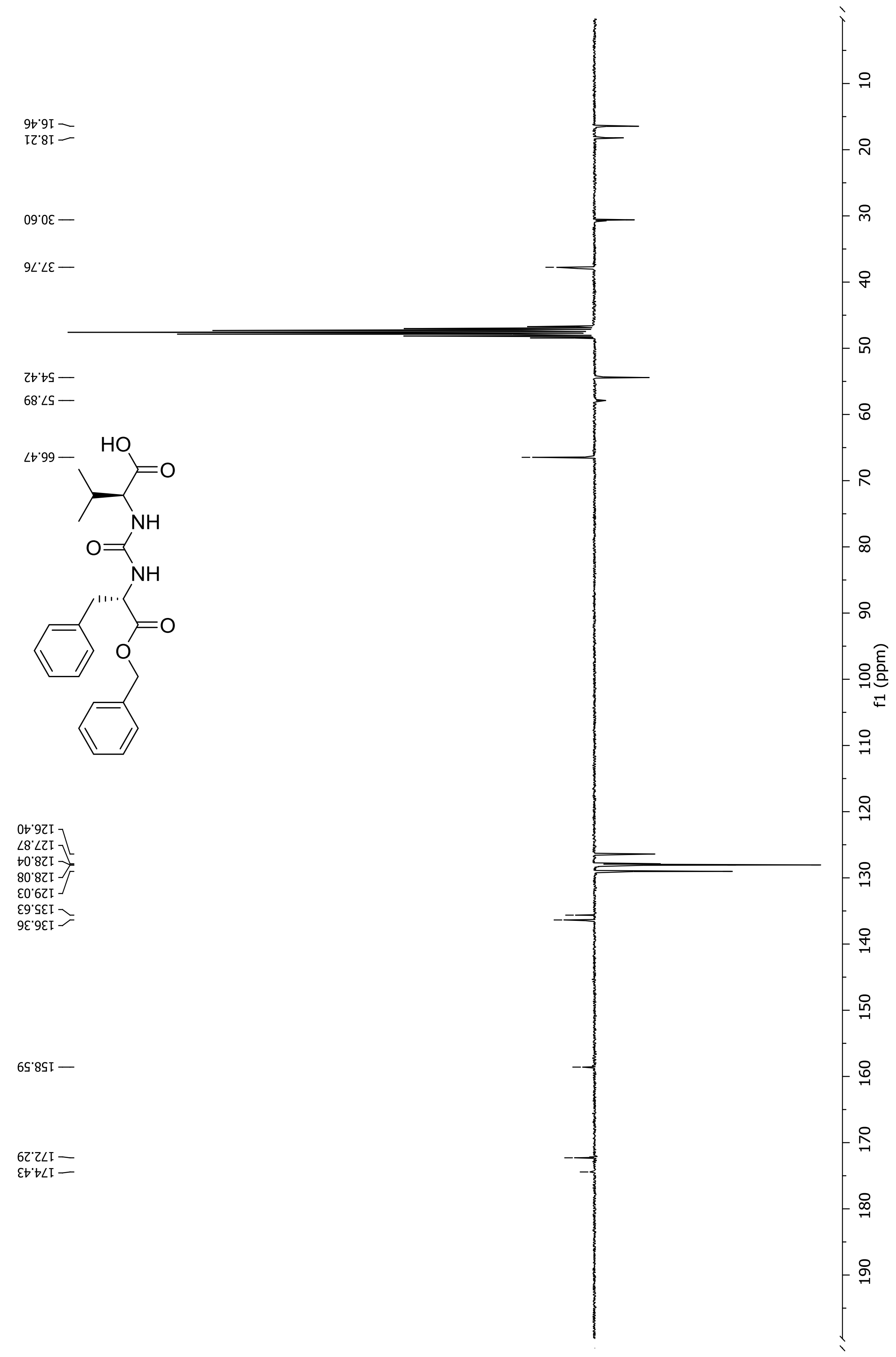

^{1}H-NMR (300 MHz, $CDCl_3$) spectrum of compound **20** (mixture of rotamers)

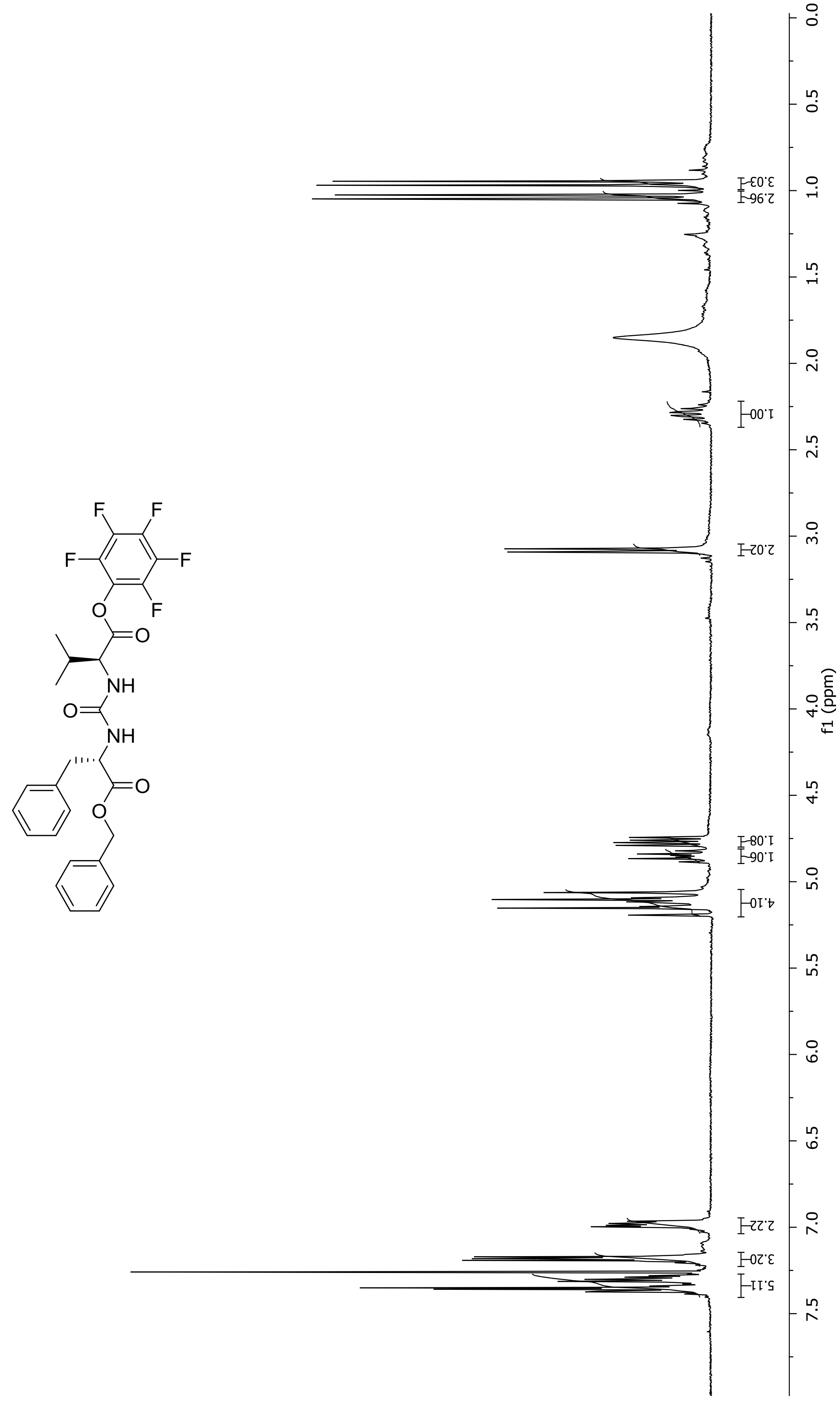

^{13}C-NMR (75 MHz, $CDCl_3$) spectrum of compound **20** (APT) (mixture of rotamers)

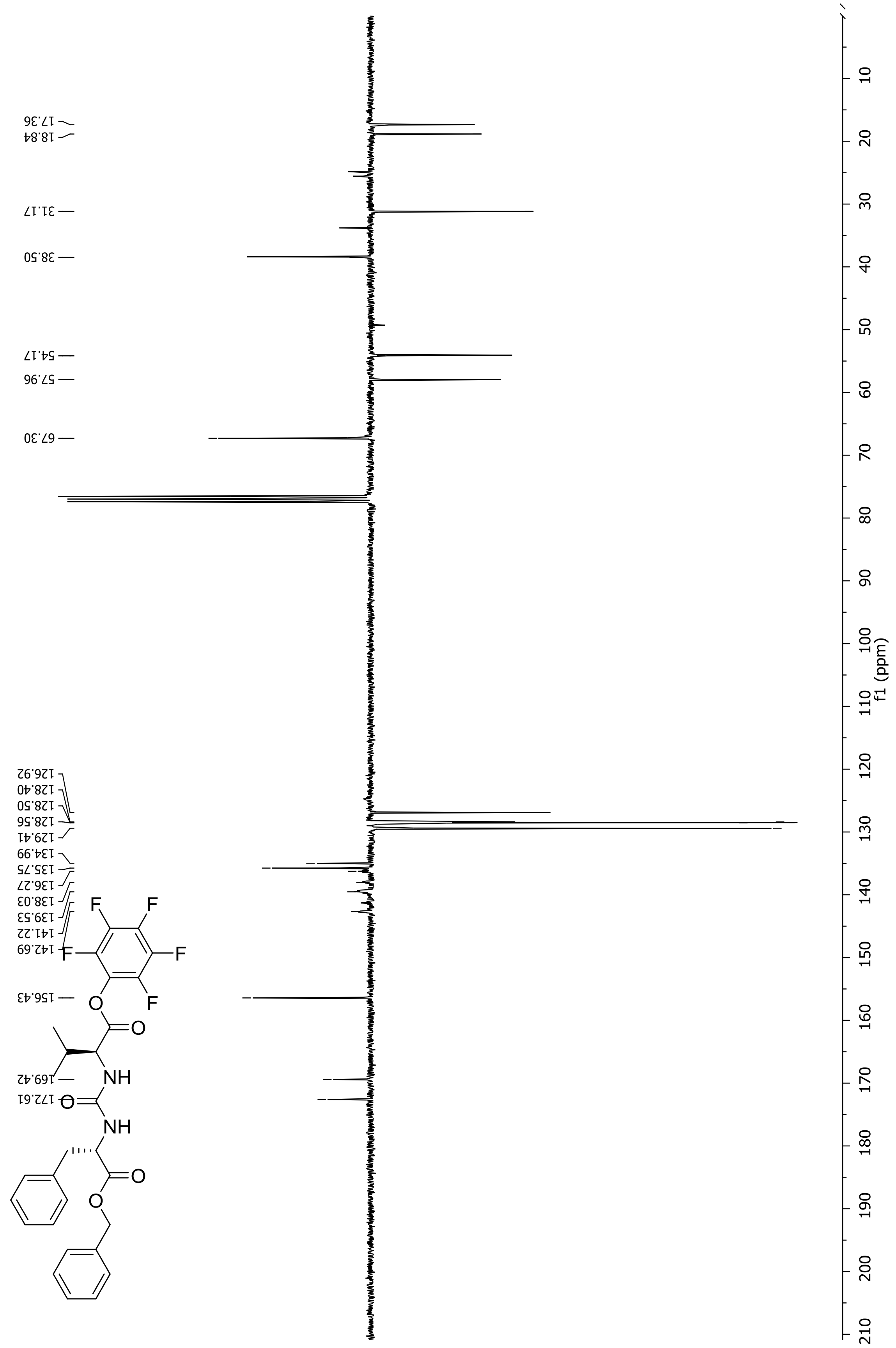

^{1}H-NMR (600 MHz, $CDCl_3$) spectrum of compound **21**

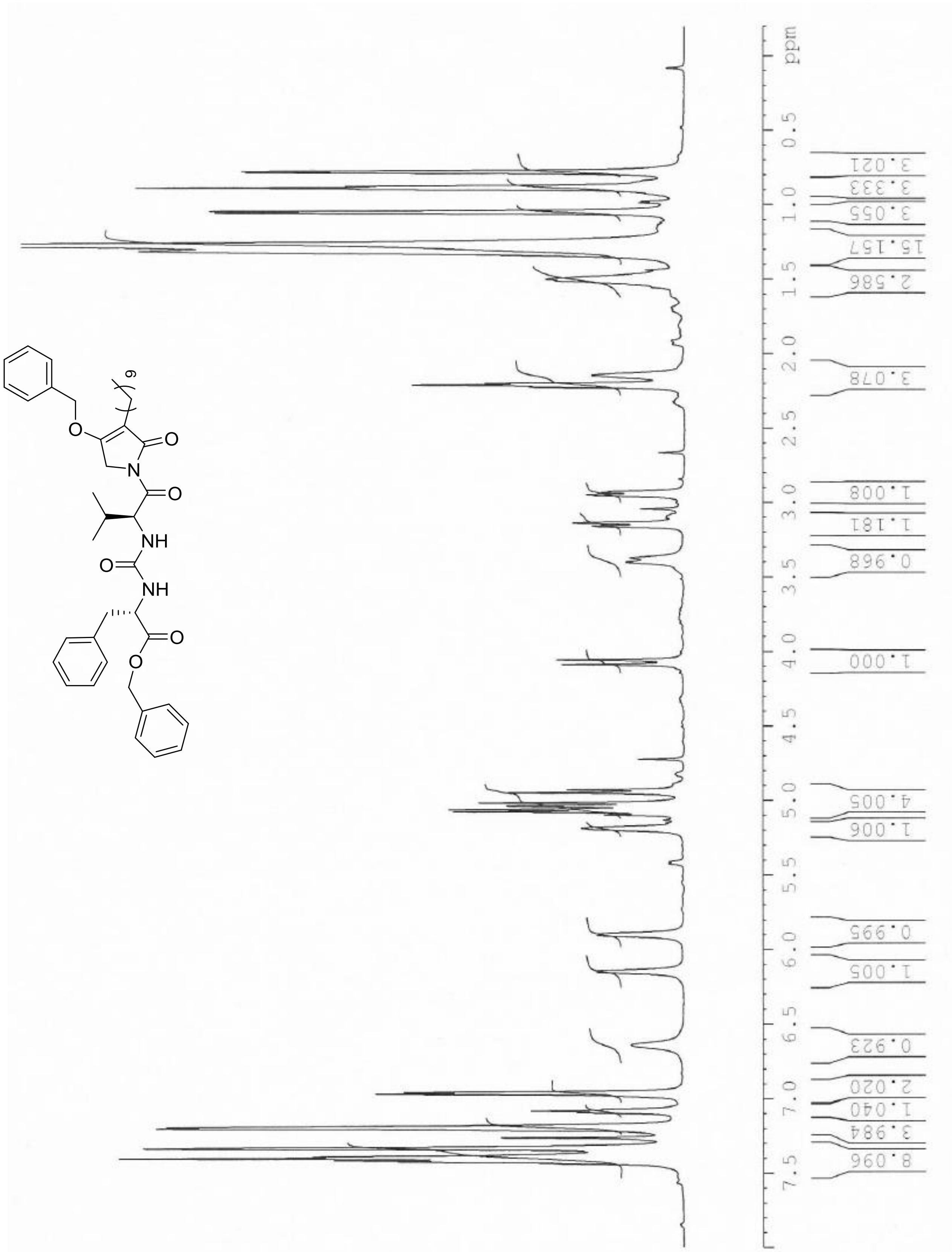

^{13}C-NMR (150 MHz, $CDCl_3$) spectrum of compound **21**

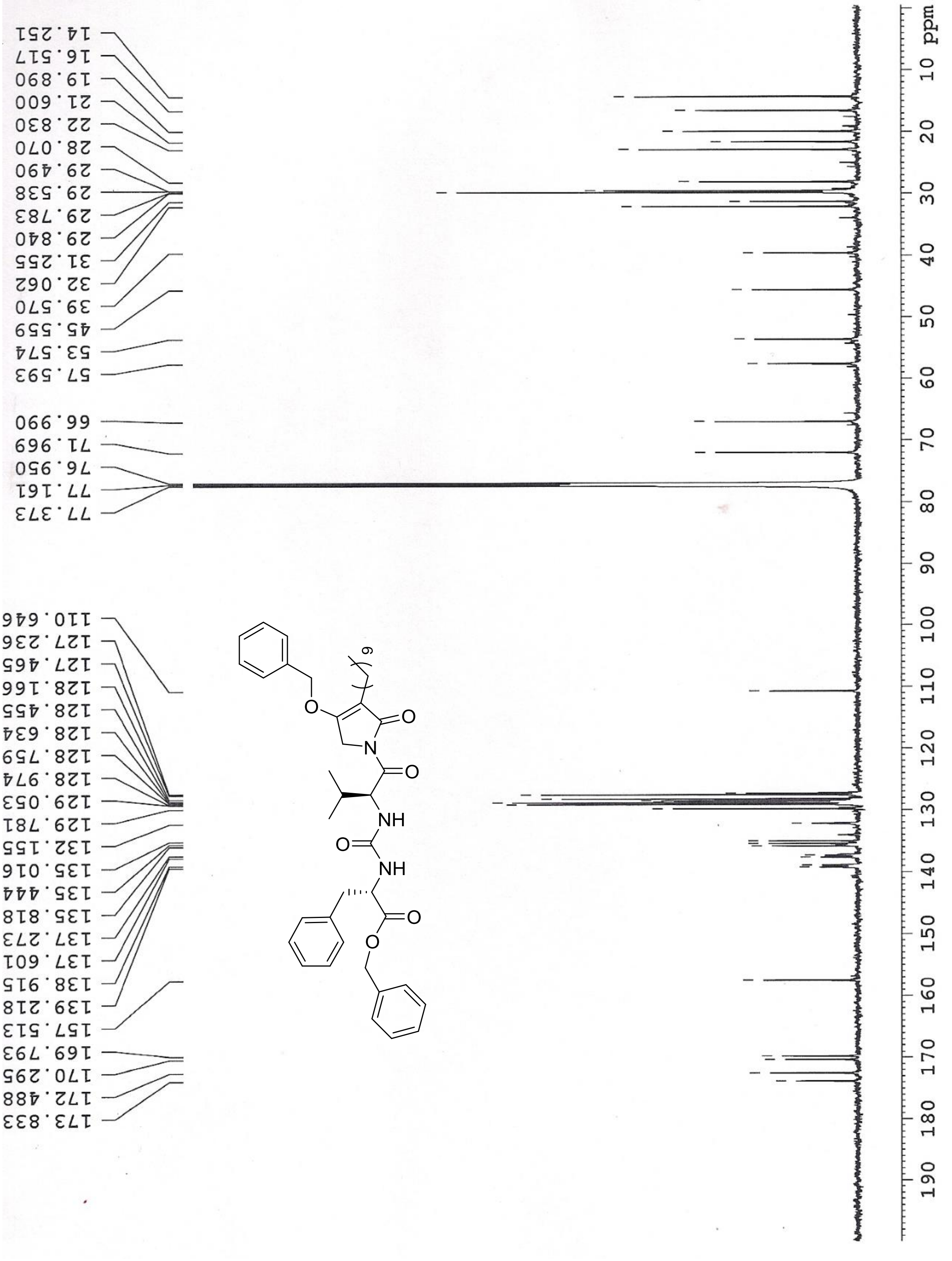

^{1}H-NMR (600 MHz, CD_3OD) spectrum of compound **1**

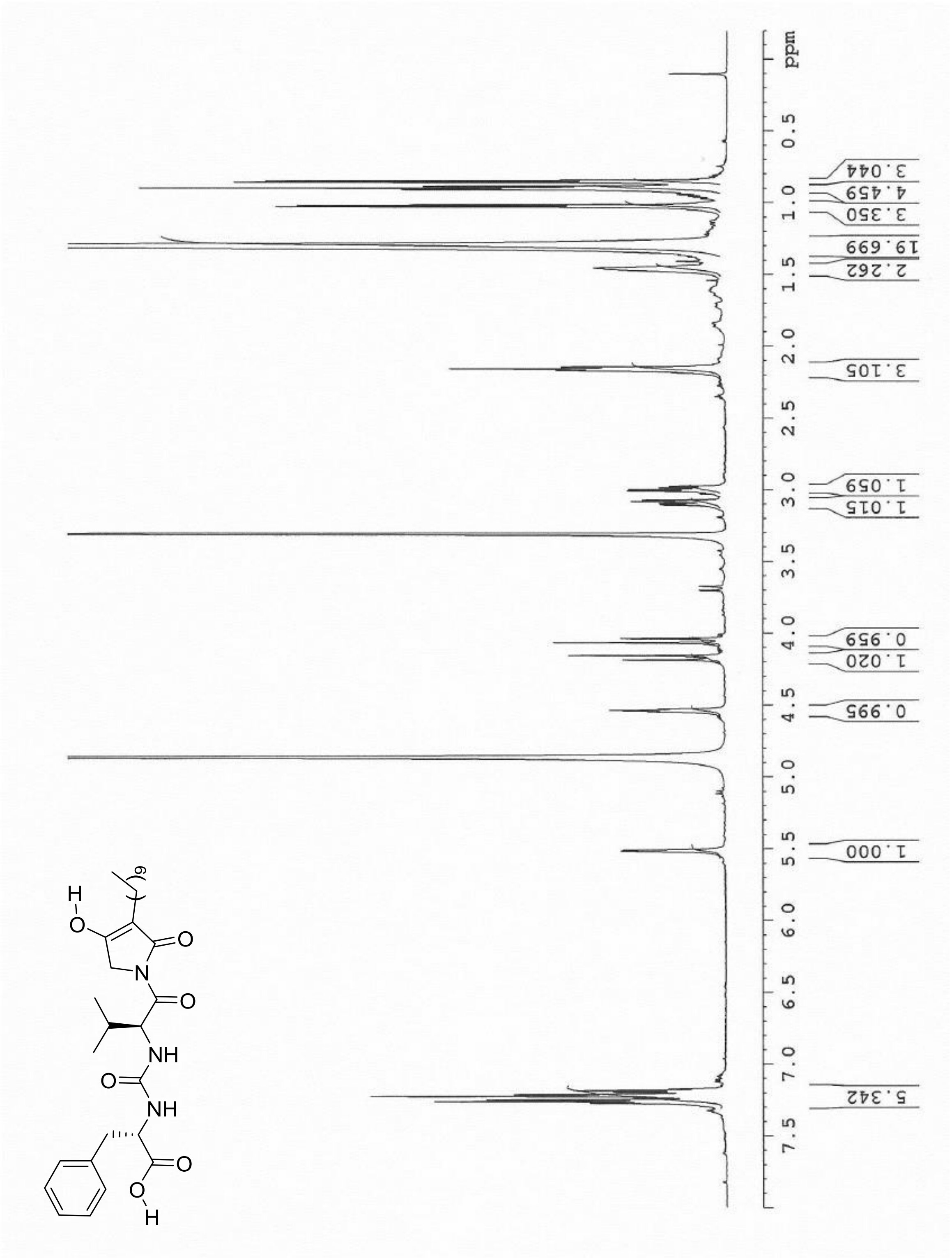

^{13}C-NMR (150 MHz, CD_3OD) spectrum of compound **1**.

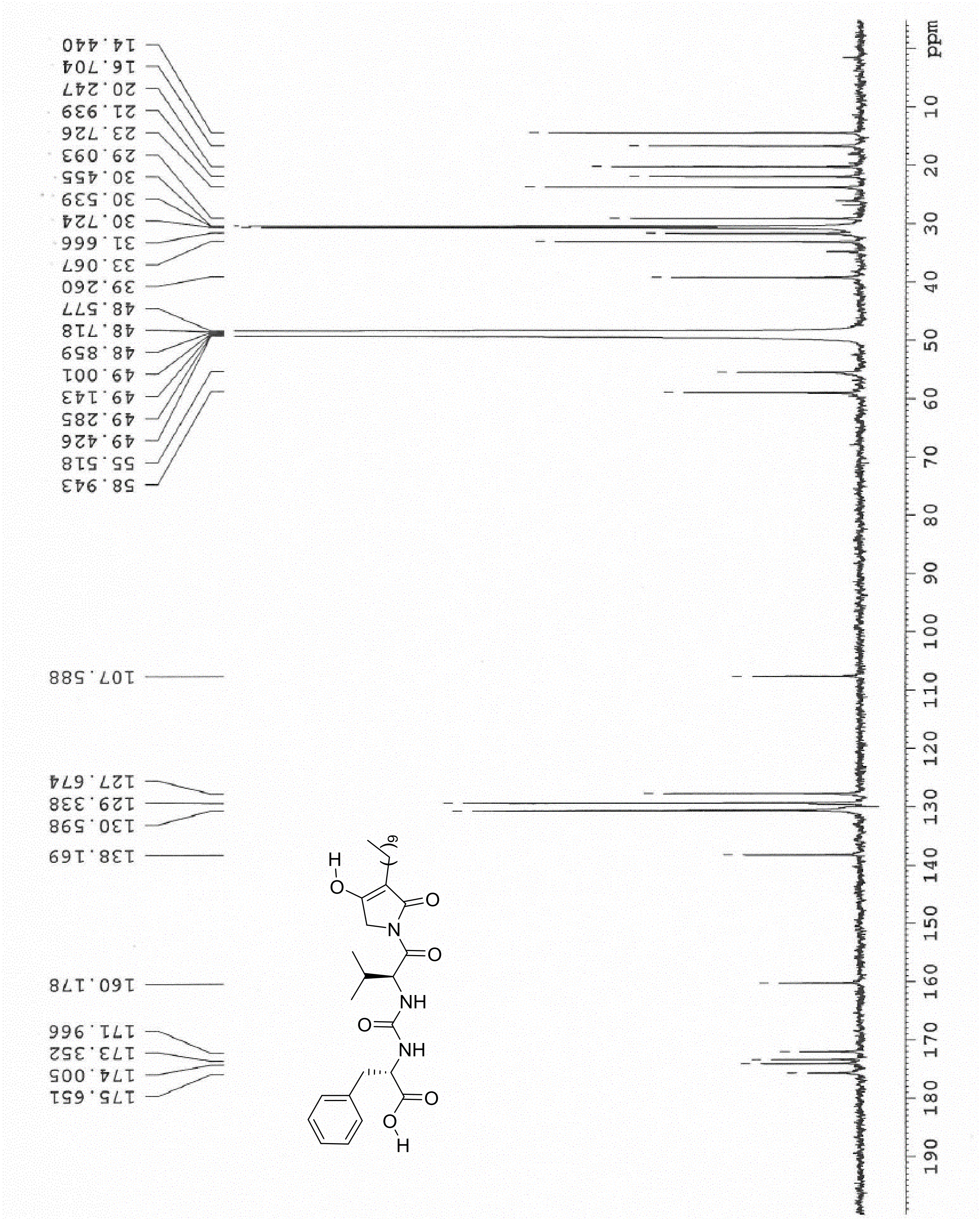

COSY (600 MHz, CD_3OD) spectrum of compound **1**.

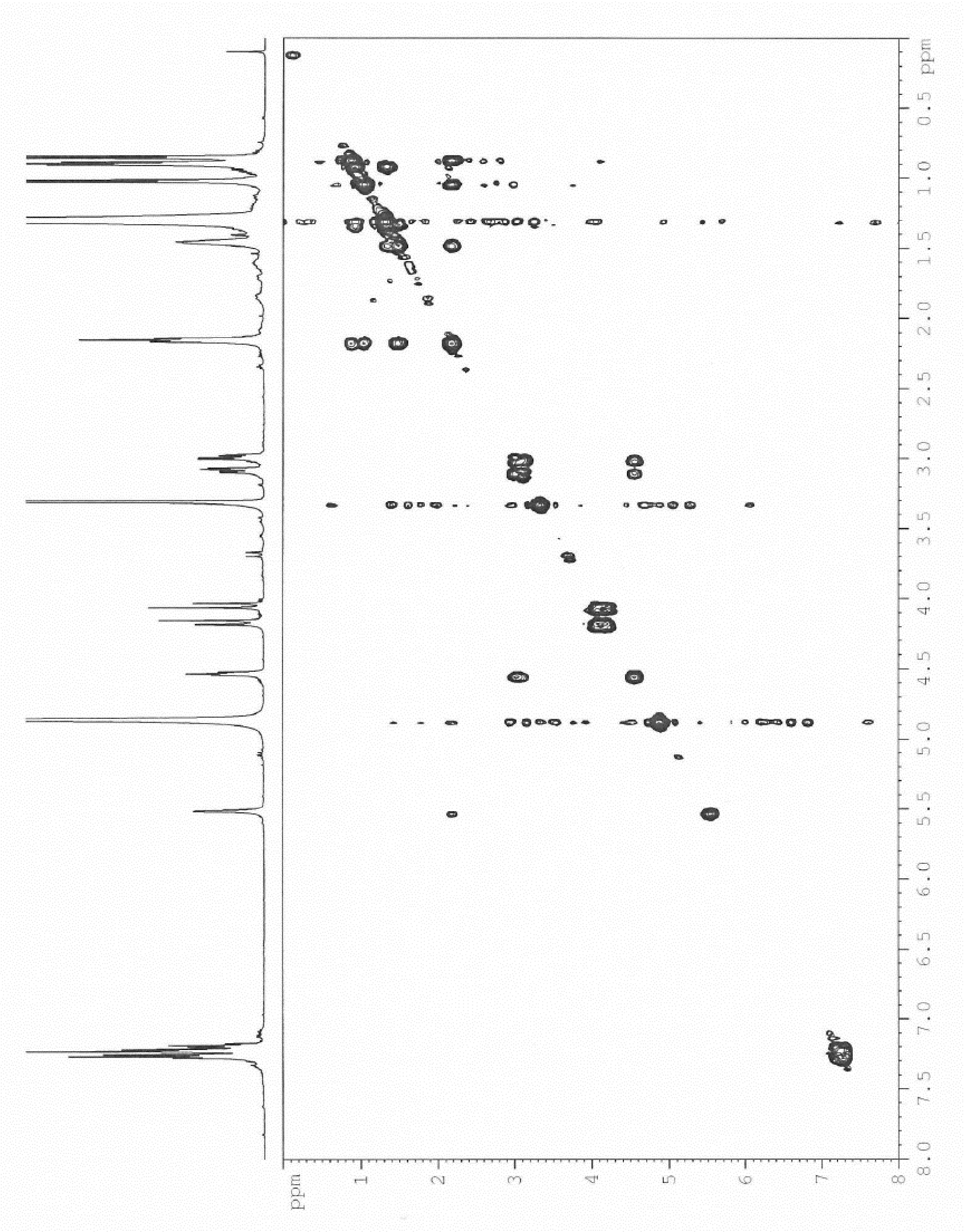

MS spectrum of compound **1**.

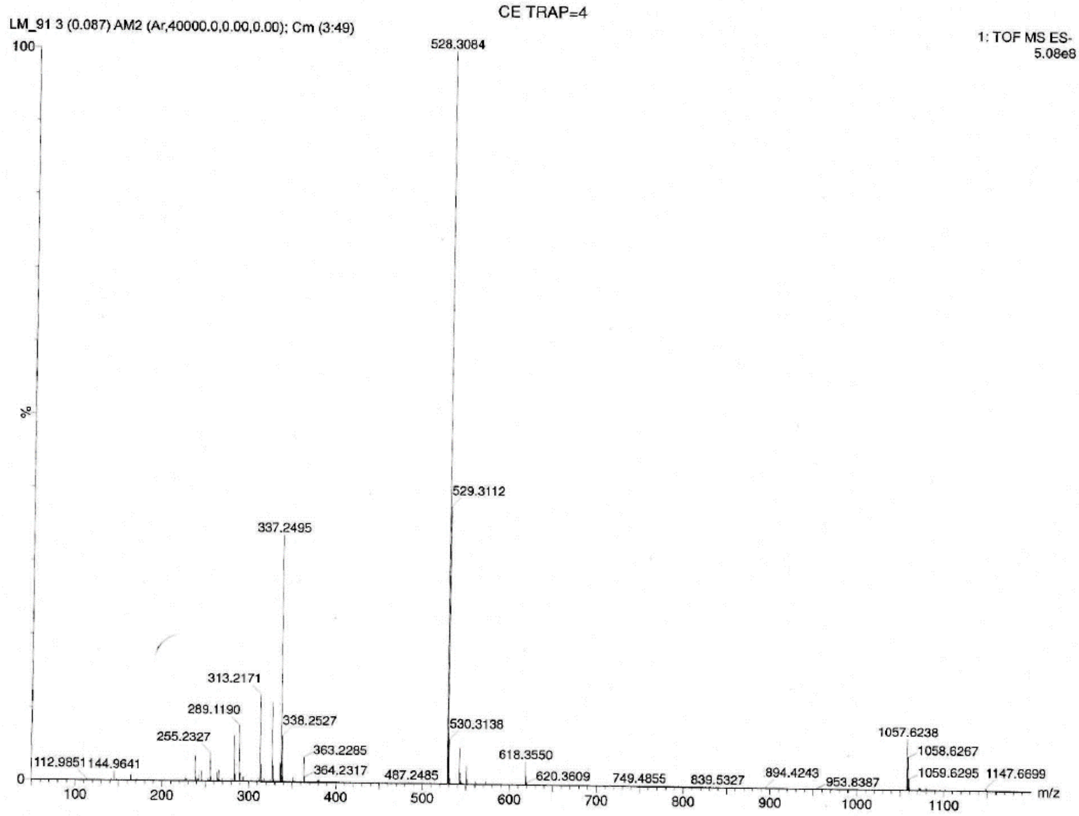

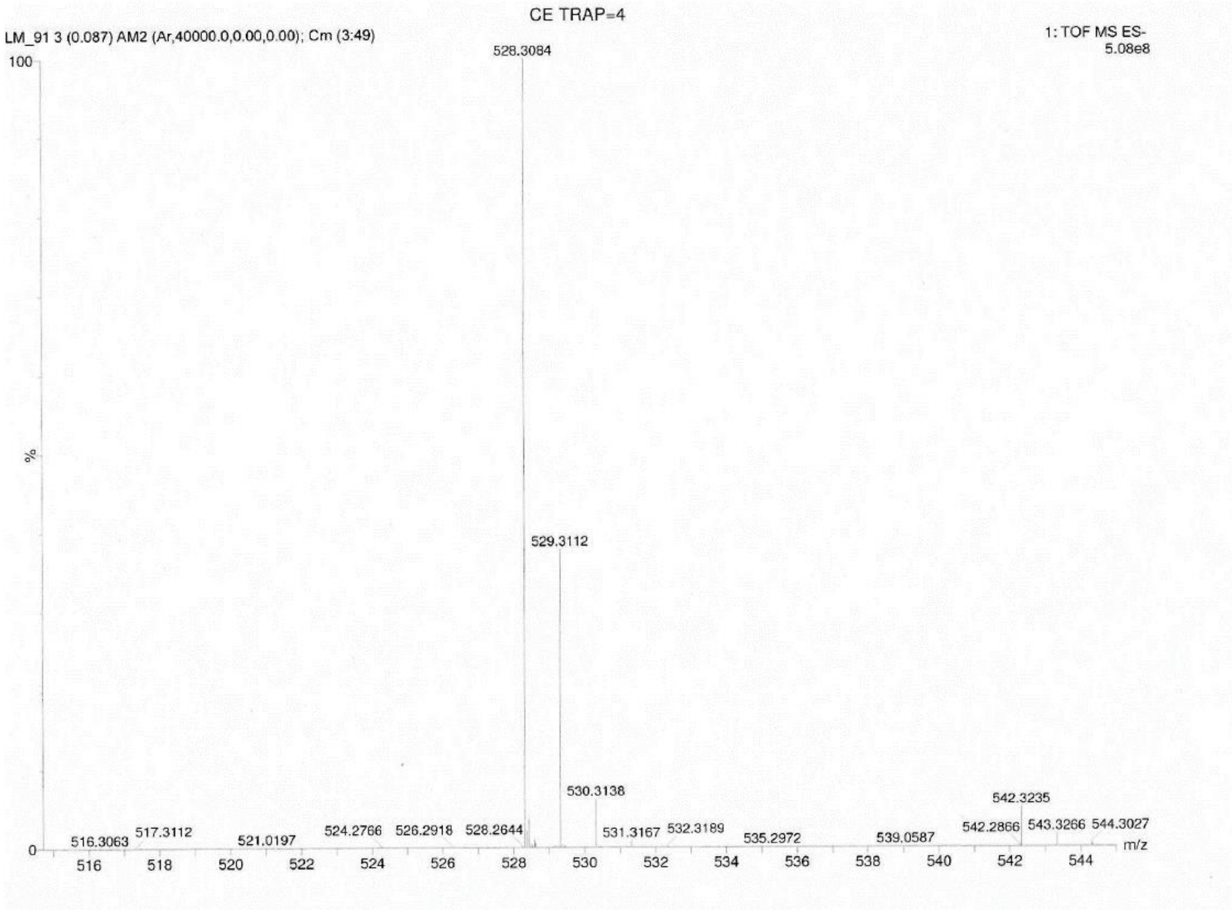

Elemental composition report of compound **1**

Elemental Composition Report — Page 1

Single Mass Analysis
Tolerance = 10.0 PPM / DBE: min = -1.5, max = 50.0
Element prediction: Off
Number of isotope peaks used for i-FIT = 9

Monoisotopic Mass, Even Electron Ions
161 formula(e) evaluated with 1 results within limits (all results (up to 1000) for each mass)
Elements Used:
C: 29-29 H: 10-100 N: 0-3 O: 0-10 Na: 0-3

CE TRAP=4 LM_91 3 (0.087) AM2 (Ar,40000.0,0.00,0.00); Cm (3.49)
1: TOF MS ES-

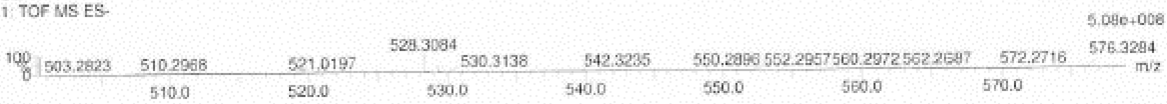

Minimum:				-1.5				
Maximum:		5.0	10.0	50.0				
Mass	Calc. Mass	mDa	PPM	DBE	i-FIT	Norm	Conf(%)	Formula
528.3084	528.3074	1.0	1.9	10.5	4738.3	n/a	n/a	C29 H42 N3 O6
